# Analysis of the FnrL regulon in *Rhodobacter capsulatus* reveals limited regulon overlap with orthologues from *Rhodobacter sphaeroides* and *Escherichia coli*

**DOI:** 10.1186/s12864-015-2162-4

**Published:** 2015-11-04

**Authors:** Joseph E. Kumka, Carl E. Bauer

**Affiliations:** Molecular and Cellular Biochemistry Department, Indiana University, Simon Hall MSB, 212 S. Hawthorne Dr, Bloomington, IN 47405-7003 USA

**Keywords:** Transcriptomics, Redox regulation, Global transcription factor binding

## Abstract

**Background:**

FNR homologues constitute an important class of transcription factors that control a wide range of anaerobic physiological functions in a number of bacterial species. Since FNR homologues are some of the most pervasive transcription factors, an understanding of their involvement in regulating anaerobic gene expression in different species sheds light on evolutionary similarity and differences. To address this question, we used a combination of high throughput RNA-Seq and ChIP-Seq analysis to define the extent of the FnrL regulon in *Rhodobacter capsulatus* and related our results to that of FnrL in *Rhodobacter sphaeroides* and FNR in *Escherichia coli.*

**Results:**

Our RNA-seq results show that FnrL affects the expression of 807 genes, which accounts for over 20 % of the *Rba. capsulatus* genome. ChIP-seq results indicate that 42 of these genes are directly regulated by FnrL. Importantly, this includes genes involved in the synthesis of the anoxygenic photosystem. Similarly, FnrL in *Rba. sphaeroides* affects 24 % of its genome, however, only 171 genes are differentially expressed in common between two *Rhodobacter* species, suggesting significant divergence in regulation.

**Conclusions:**

We show that FnrL in *Rba. capsulatus* activates photosynthesis while in *Rba. sphaeroides* FnrL regulation reported to involve repression of the photosystem. This analysis highlights important differences in transcriptional control of photosynthetic events and other metabolic processes controlled by FnrL orthologues in closely related *Rhodobacter* species*.* Furthermore, we also show that the *E. coli* FNR regulon has limited transcriptional overlap with the FnrL regulons from either *Rhodobacter* species.

**Electronic supplementary material:**

The online version of this article (doi:10.1186/s12864-015-2162-4) contains supplementary material, which is available to authorized users.

## Background

The purple non-sulfur α-proteobacterium *Rhodobacter capsulatus* possesses a metabolically versatile metabolism that allows growth in a wide variety of environments. Much is known about its photosynthetic growth metabolism along with transcription factors that control anaerobic photosystem gene expression such as RegA, CrtJ, and AerR [1–5]. However, the redox responding transcription factor FnrL, which is a homologue of FNR (for fumarate nitrate reduction) from *E. coli,* has not been well characterized in *Rba. capsulatus* [5–7]. FnrL from *Rba. capsulatus* is reported to have a role in production of respiratory cytochromes but not in the production of the photosystem machinery [2, 5, 7, 8]. Beyond these observations, the involvement of FnrL in controlling anaerobic gene expression is unknown.

FNR from *E. coli* has a central role in controlling many changes in metabolism that occurs when these cells shift from aerobic to anaerobic growth conditions [6, 9]. FNR directly senses changes in oxygen tension via the presence of a redox sensitive 4Fe-4S cluster that is coordinated by four cysteines [10]. Under anaerobic conditions, the iron cluster is stable allowing FNR to form a dimer that binds to target DNA sequences [11, 12]. However, under aerobic conditions, this cluster becomes oxidized leading to its disassembly with a concomitant loss of FNR dimerization and ultimately loss of DNA binding activity [8, 11]. FnrL from *Rhodobacter capsulatus,* and its homolog in *Rhodobacter sphaeroides,* also contain four Fe coordinating cysteines as described for *E. coli* FNR, however their placement within the peptide sequence is different from FNR. This suggests that the coordination of the 4Fe-4S cluster may be altered and/or there exist dissimilarities in redox regulation and allosteric behavior between the FnrL homologs and FNR.

Analysis of the FNR regulon in *E. coli* has been well characterized most recently using a combination of the deep sequencing technologies; RNA-seq and chromatin immunoprecipitation sequencing (ChIP-seq) [6]. This recent study has established that the FNR regulon is quite large and complex and is responsible for controlling variety of genes that affect the ability to effectively grow under conditions of oxygen limitation. For example, FNR controls the expression of high oxygen affinity terminal oxidases and a DMSO reductase that uses DMSO as an alternative electron acceptor under anaerobiosis [6]. The FNR regulon not only includes genes whose expression are directly regulated by FNR, but also genes indirectly regulated by FNR via secondary regulation [6, 13]. The latter occurs when FNR directly controls the expression of a transcription factor that subsequently regulates expression of downstream genes either directly or through additional downstream transcription cascades. Analysis of the *E. coli* FNR regulon is further complicated by the observation that a number of FNR binding sites as defined by ChIP-seq occur near or within genes that do not exhibit a corresponding difference in expression upon deletion of FNR [6]. Thus, there appears to be a number of “silent” FNR binding sites that presumably are involved in control of gene expression under conditions that have not yet been tested. Additionally, these silent sites may have a role that does not affect transcription but instead have a role in providing chromosomal structural integrity. For example, FNR may have a yet to be defined nucleoid-associated role that would affect such processes as chromosome packing [14].

Both RNA-seq and ChIP-seq analysis of the *Rba, sphaeroides* FnrL regulon has recently been reported [18]. Their analysis indicated that FnrL is directly involved in regulating anaerobic respiration, tetrapyrrole biosynthesis and iron metabolism. However, there does not appear to be direct control of the photosynthetic structural proteins with overall photosynthetic events negatively regulated by FnrL. In contrast, detailed analysis of the *Rba. capsulatus* FnrL regulon has not been undertaken, but is necessary as there are key differences between the observed phenotypes of FnrL deletions in these species*.* For example, FnrL mutants in *Rba. sphaeroides* are unable to grow photosynthetically while a FnrL deletion mutant of *Rba. capsulatus* remains viable during photosynthetic growth [5, 7, 15–17]. To address these differences, we utilize a combination of ChIP-seq and RNA-seq analyses to provide a high-resolution description of the FnrL regulon in *Rba. capsulatus*. We have identified a large set of genes scattered throughout the genome involved in diverse metabolic pathways that are directly and indirectly regulated by FnrL. We present a global picture of the regulatory involvement of FnrL and also provide a detailed depiction of the photosynthetic events controlled by FnrL in *Rba. capsulatus.* For completeness, we compare the *Rba. capsulatus* FnrL regulon with the FnrL regulon from *Rba. sphaeroides* and the FNR regulon in *E. coli* [6, 18]. While the FnrL regulons from *Rhodobacter* species do share similarities, they differ significantly and are unambiguously different from the *E. coli* FNR regulon. Consequently, there is considerable plasticity in number and type of genes that constitute members of FNR regulons in different organisms.

## Results and discussion

### Identifying direct and indirect members of the FnrL regulon using comparative RNA-Seq and ChIP-Seq

**Fig. 1:**
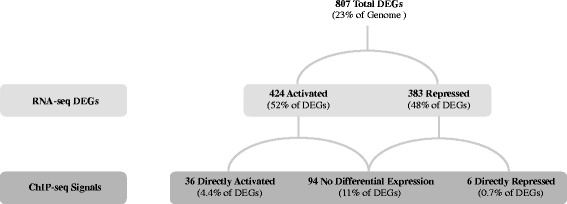
Total FnrL directly and indirectly controlled genes. Differentially expressed genes that are directly and indirectly activated by FnrL as determined from RNA-seq (green) and ChIP-seq (purple). No Differential Expression are instances where ChIP signal was observed without a corresponding RNA-seq expression

We identified members of the FnrL regulon by performing RNA-seq transcriptome analysis of anaerobically (photosynthetically) grown wild-type versus Δ*fnrL* strains. Over 10 million (M) strand specific RNA-seq reads were collected per sample from three biological replicates. Differentially expressed genes (DEGs) from pair-wise comparison of wild type and Δ*fnrL* data sets were identified as those that had altered photosynthetic/aerobic changes in expression with a *p*-value ≤ 0.05. The motivation behind using a *p*-value cutoff of ≤ 0.05 was to make our results directly comparable to that of previously published *E. coli* and *Rba. sphaeroides* FNR/FnrL RNA-seq data sets that used a similar *p*-value of ≤ 0.05 [6, 18]. With a *p*-value cutoff of ≤ 0.05 we categorized 807 DEGs as members of the *Rba. capsulatus* FnrL regulon (Fig. 1, Table 1, Additional files 1 and 2: Table S1 and S2). This number of genes in the *Rba. capsulatus* FnrL regulon is comparable to that observed for the FnrL regulon from *Rba. sphaeroides,* which has 917 genes DEG’s with *p*-value ≤ 0.05 We also note that several FnrL ChIP-seq peaks containing well-defined FnrL binding consensus sequences are present upstream of DEGs with *p*-values between 0.05 and 0.1. These genes are noted in the ChIP-seq data set in Additional file 3: Table S3 and suggest that a *p*-value ≤ 0.05 at times acts as too stringent of a filter. Nevertheless we used the *p* value ≤ 0.05 as a cut off so as to be confident that the genes that are included in the FnrL regulon are not falsely identified and to be consistent with similar studies in other species.

**Table 1:** FnrL directly regulated genes based on ChIP-seq signal with corresponding RNA-seq expression change that also contain a consensus binding sequence

Locus ID	Gene Name	Description	Recognition Sequence	Enrichment	Regulation	Fold Change
COG C: Energy production and conversion						
RCC01157	*ccoN*	*cbb*_*3*_-type cytochrome c oxidase subunit I	TTGATCAAGGTCAA^b^	25	+	1.55
RCC01157	*ccoN*	*cbb*_*3*_-type cytochrome c oxidase subunit I	ATGATGTCGATCAA^a^	25	+	1.55
RCC00728	*NnrU family protein*	NnrU family protein	CTGCCGCAGATCAA^a^	4	+	1.47
RCC00732	*sdhD*	succinate dehydrogenase	ATGATGAGCGTCAA^b^	3	+	1.41
RCC00022	*Oxidoreductase*	oxidoreductase	ATGATTTACCGCAA^a^	5	+	1.38
COG E: Amino acid transport and metabolism						
RCC01724	*speB1*	agmatinase	TTGATCTGCGTCAA^b^	10	+	1.33
COG F: Nucleotide transport and metabolism						
RCC00400	*pyrB*	aspartate carbamoyltransferase	CTGACGCAGATCAA^a^	10	+	1.47
COG G: Carbohydrate transport and metabolism						
RCC00731	*sdhC*	succinate dehydrogenase, cytochrome b556 subunit	ATGATGAGCGTCAA^a^	3	+	1.53
COG I: Lipid transport and metabolism						
RCC00480	*rpsU*	30S ribosomal protein S21	CTGATGCAACTCAA^b^	4	+	1.57
COG J: Translation, ribisomal tructure and biogenesis						
RCC01495	*fusA*	translation elongation factor G	TTGGCATGGGTCAA^b^	17	+	3.48
COG L: Replication, recombination and repair						
RCC03240	*ATPase AAA*	K01144 exodeoxyribonuclease V	ATGCGCCAGATCAA^a^	4	-	-1.34
RCC02193	*DNA-3-methyladenine glycosylase II*	K01247 DNA-3-methyladenine glycosylase II	ATGACGCGGATCAA^a^	4	-	–1.97
COG M: Cell wall/membrane/envelope biogenesis						
RCC02479	*lipoprotein*	lipoprotein	CTGATGCAGCGCAA^b^	13	+	1.42
COG N: Cell motility						
RCC00481	*mcpI*	methyl-accepting chemotaxis protein McpI	CTGACCGAGATCAA^a^	4	-	–1.53
RCC03524	*flagellar FlaF family protein*	K06602 flagellar protein FlaF	CTGATCGACATCAA^a^	4	-	–1.87
RCC03523	*flbT*	flagellin synthesis repressor protein FlbT	CTGATCGACATCAA^b^	4	-	–2.14
COG O: Post-translational modification, protein turnover, and chaperones						
RCC01156	*UspA domain-containing protein*	UspA domain-containing protein	TTGACGCGGATCAA^b^	26	+	5.21
RCC01723	*ccpA*	cytochrome-c peroxidase	TTGATCTGCGTCAA^b^	10	+	3.81
RCC02829	*hypothetical protein*	K03699 putative hemolysin	TTGACCCTCGTCAA^a^	6	-	–1.64
COG R: General function prediction only						
RCC02684	*polyphosphate kinase 2*	polyphosphate kinase 2	TTGATGCGTGTCAA^b^	14	+	2.25
RCC02665	*hemolysin-type calcium-binding*	hemolysin-type calcium-binding	ATGACCGGCGTCAA^a^	9	+	1.46
COG S: Function unknown						
RCC00435	*hypothetical protein*	hypothetical protein	CTGACCCAGATCAA^b^	21	+	9.19
RCC00901	*hypothetical protein*	hypothetical protein	TTGACACGGGTCAA^b^	10	+	6.87
RCC00747	*hypothetical protein*	hypothetical protein	ATGACGCAGATCAA^b^	5	+	3.78
RCC00424	*hypothetical protein*	hypothetical protein	ATGATTCAGATCAA^b^	20	+	3.51
RCC02321	*hypothetical protein*	hypothetical protein	ATGATCCGGATCAA^b^	26	+	2.17
RCC02988	*hypothetical protein*	hypothetical protein	TTGACCCAGATCAA^b^	8	-	–1.41
RCC01027	*hypothetical protein*	hypothetical protein	TTGACCAAGGTCAA^b^	6	-	–1.64
COG T: Signal transduction mechanisms						
RCC02849	*dorS*	DMSO/TMAO-sensor hybrid histidine kinase	TTGATCGGGATCAA^a^	26	+	4.53
RCC02590	*dksA*	DnaK suppressor protein	TTGATTCAGGTCAA^b^	28	+	1.79
COG X: Photosynthesis						
RCC00667	*aerR*	regulatory CrtJ antirepressor AerR	ATGCTCGAGTTCAA^b^	8	+	1.39
RCC00666	*bchF*	2-vinyl bacteriochlorophyllide hydratase	ATGACATGGGTCAA^b^	8	+	1.39

Recognition sequences were determined using MEME server

^a^Sequence is found within the coding region of the gene

^b^Sequence is found in the upstream intergenic or promoter region

We determined which DEGs are directly controlled by FnrL by identifying FnrL binding sites *in vivo* using ChIP-seq analysis. Our ChIP-seq results provided near-complete representation of the entire genome with significant peaks called that exhibited a false discovery rate (FDR) cutoff of 5 % (corresponding to an unadjusted *p* value <1E-5) using the MACS package. In making our results comparable to datasets available for *E. coli* and *Rba. sphaeroides,* we present FDR values with a cutoff of 5 %. As shown in Additional file 1: Table S1 we identified 82 ChIP-seq peaks that were above this significance threshold. These peaks were found primarily within the intergenic regions where 47 ChIP sites (57 %) are enriched in promoter regions and of these 28 show a corresponding differential expression. Using chi-squared test it was determined that this exhibits statistical enrichment for promoters since intergenic regions only make up 9.19 % of *Rba. capsulatus’* genome. Furthermore, we also identified peaks that were located within a gene next to neighboring genes that exhibited differential gene expression in the Δ*fnrL* strain (12 cases). We also found 34 called FnrL ChIP-seq peaks that did not exhibit an alteration in neighboring gene expression (Additional file 1: Table S1). It is difficult to reconcile the possibility that the latter category represents false positives on the basis of excellent enrichment coupled with a clear FnrL recognition sequence; rather, it may signal that FnrL bound to these location either has long range expression effects that are not being recognized or that additional auxiliary regulatory factors supersede the activity of FnrL. Furthermore, since only the photosynthetic state was investigated, these binding sites may be important in gene regulation during other growth states such as dark anaerobic or microaerobic growth or under nutrient limiting conditions.

**Fig. 2:**
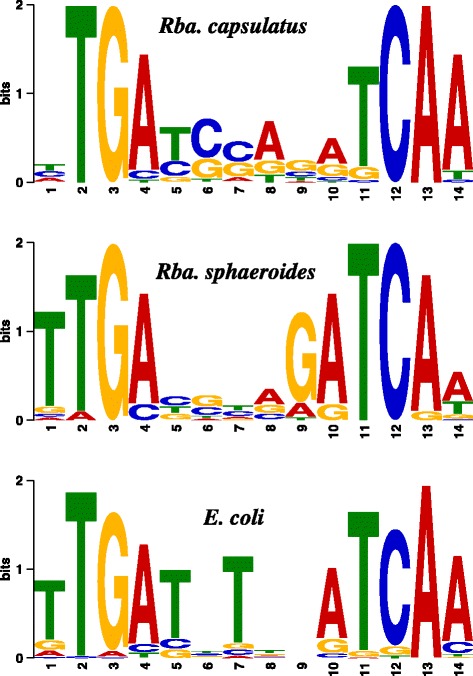
DNA binding motifs of FnrL/FNR orthologues. DNA consensus binding site was determined using MEME server from ChIP enriched sequences.

A consensus FnrL recognition sequence was obtained using the MEME server from called ChIP-seq sites (Fig. 2). The derived sequence (T/C/A)TGA-N6-TCAA has second and third positions that were invariably TG while the 12th and 13th positions were invariably CA. The first position was somewhat variable with T, C, or A accounting for 37, 34 and 24 %, respectively, whereas the 14th position was an A at a frequency of 90 %. As shown in Fig. 2, the derived FnrL consensus sequence is highly similar to consensus sequences derived from similar studies from *Rba. sphaeroides* and *E. coli*. Variants of the *Rba. capsulatus* FnrL recognition sequence were identified by MEME in 69 out of 82 called ChIP-seq sites (Additional file 1: Table S1) with potential FnrL binding recognition sequences also manually found in ChIP peaks where no consensus sequence was identified by MEME. These manually identified potential recognition sequences are not listed in Additional file 1: Table S1 since flanking TTG/CAA sequences are common throughout the genome.

We also screened the *Rba. capsulatus* genome for additional FnrL sites with Virtual Footprint using FnrL recognition sequences identified from ChIP-seq peaks [19]. Our motivation for this stemmed from the fact that technical limitations exist that likely limit effective *in vivo* crosslinking of FnrL and/or immunoprecipitation of crosslinked DNA segments thus prohibiting our ability to identify all sites that are bound with FnrL. For example, we utilized formaldehyde as a crosslinker as it is typically used for ChIP-seq analysis. However, formaldehyde is known to form an ineffective adduct with B-form double stranded DNA and is thought to only be an effective crosslinker in cases where DNA binding proteins have perturbed or melted the DNA structure to allow formaldehyde to interact with the amine group of adenine [20]. Therefore, it is conceivable that FnrL bound to some sites may be ineffectively crosslinked with formaldehyde. Consequently the additional screening for potential FnrL sites using the MEME identified recognition sequences not surprisingly resulted in the identification of 332 additional potential FnrL recognition sites for a total of 414 possible sites in the genome. These additional sites were subsequently analyzed for their location relative to FnrL dependent differential gene expression. From this analysis, we were able to determine that an additional 77 genes are likely under direct control of FnrL as evidenced by the presence of a putative FnrL recognition site near a differentially expressed gene (Additional file 4: Table S4). Note that even thought some of these additional genes are likely directly regulated by FnrL they have remained in the “indirectly regulated” category (Additional file 2: Table S2) as it will require additional experimentation to determine which of genes are indeed under direct control by FnrL.

### COG assignment of the FnrL regulon members

**Fig. 3:**
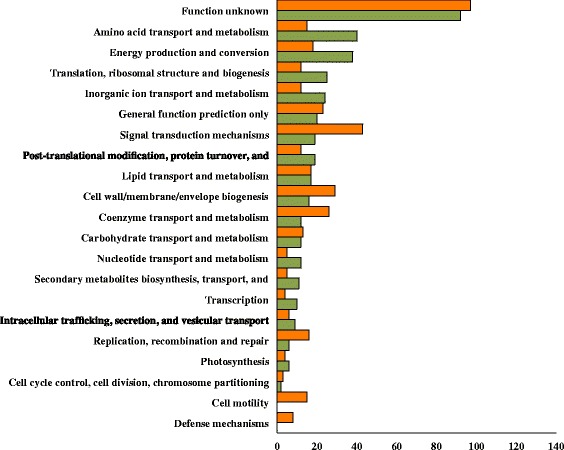
*Rba. capsulatus* genes clustered based on orthologous groups/functions. All FnrL directly and indirectly controlled genes clustered based on orthologous groups with orange representing repressed and green activated gene counts. COGs were determined using eggNOG server

To address the role of members of the FnrL regulon in controlling anaerobic physiology, we placed individual genes into different “Clusters Of Orthologous Groups” (COGs) (as categorized in Additional file 5: Table S5. Inspection of the bar chart in Fig. 3 shows that the largest set of genes directly controlled by FnrL are in the category “Function Unknown”, which, accounts for 27 % of the genes in this regulon. This underscores that the role of many gene products in microbial physiology remain to be discovered. The largest COG categories that have a defined function are “Amino Acid Transport and Metabolism” and “Energy Production and Conversion”. These major COG categories highlight that FnrL has a role in controlling the energy metabolism of these cells. Another major category is “Signal transduction” of which more genes are repressed than activated. Signal transduction, along with the COG category “Transcription”, underscores that FnrL is an overarching global regulator that indirectly regulates a large number of genes.

### FnrL regulates a variety of transcription factors and signal transduction components

Analysis of regulatory proteins that are directly regulated by FnrL shows that MerR (rcc03147) and TetR (rcc03059) transcription factor family members are directly repressed by FnrL (Additional file 4: Table S4). There is also a ChIP-seq identified FnrL binding site located directly upstream of a BadM/Rf2 family regulator (Additional file 1: Table S1). FnrL also directly regulates several two-component signal transduction components. For example, FnrL binds upstream of three sensor histidine kinases coded by *rcc03452*, *rcc02198*, and RegB2 (*rcc01026*). RegB2 is divergently transcribed from its cognate response regulator partner RegA2 so FnrL may control expression of both signaling components with the caveat that no affect of deleting FnrL was observed on RegB2 and RegA2 expression under the assayed growth conditions. The physiological role of RegB2, RegA2 is unknown, but they do share some degree of similarity (28 and 44 %) to RegB/RegA system, which is a well-characterized redox response system in *Rba. capsulatus*.

**Fig. 4:**
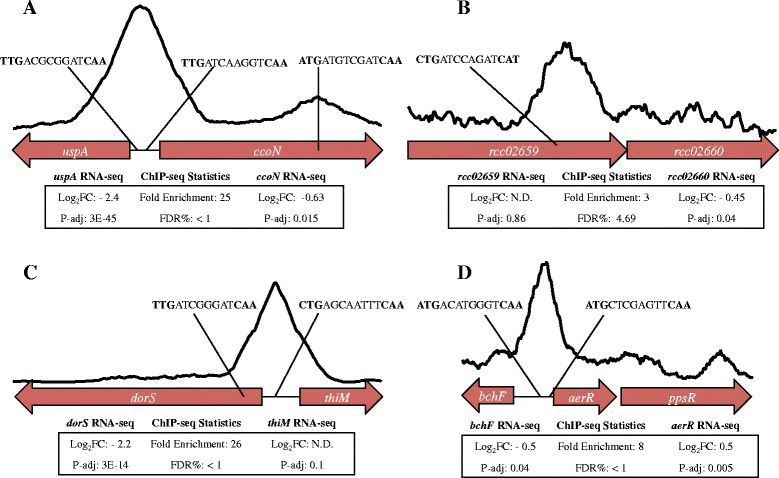
Selected ChIP and RNA-seq signal profile and statistics. Selected FnrL ChIP-seq signals of, A, cytochrome *cbb*_*3*_ promoter region, B, ABC transporters rcc02659/rcc02660 with low enrichment but one with an FnrL binding site and a corresponding differential expression based on RNA-seq, C, promoter region of DMSO histidine kinase for DMSO reductase induction, D, bacteriochlorophyll biosynthesis *bchF* and CrtJ anti-repressor *aerR* for photosynthetic induction

A two-component histidine kinase (*rcc02198*) is also a direct member of the FnrL regulon with its presumed cognate transcription response regulator (*rcc02197*) immediately upstream. These regulators are next to a propanediol gene cluster and may have a function in propanediol metabolism. The DNA binding site is located in the intergenic region of *rcc02198-rcc02199* thus only *rcc02198* is counted in the direct FnrL regulon. The ChIP-seq peak is located 185 bp upstream of the histidine kinase coding region with a corresponding 2-fold difference in transcription expression (Fig. 4c). We also observed that expression of DorS is induced 4-fold by FnrL with the presence of a ChIP-seq peak upstream of DorS, which is required for activation of the *torCAD* operon that codes for the DMSO/TMAO reductase system. It has been reported that a deletion of FnrL leads to a defect in utilizing DMSO as a terminal electron acceptor [16].

Finally, FnrL also directly activates several genes that control synthesis and or hydrolysis of di-c-GMP (*rcc02540, rcc01110* and *rcc00783*), which is often involved in regulating motility and biofilm biosynthesis suggesting that FnrL also has a role in controlling these processes [21].

### FnrL is a direct controller of anaerobic respiration and photosynthesis

Cytochrome *cbb*_*3*_ (*ccoNOQP*) appears to be under direct control of FnrL. A ChIP-seq peak was found containing an FnrL binding sequence 100 bp upstream of the *ccoN* start codon and a second recognition site within the *ccoN* gene (Fig. 4a). RNA-Seq indicates that FnrL up-regulates expression of the *ccoNOQP* operon 1.5-fold under photosynthetic conditions. This is peculiar since this operon is repressed by several additional redox regulators such as by RegA [5, 7, 22]. One explanation might be that significant FnrL activation of the divergently transcribed neighbor *uspA*, overpowers FnrL repression of *ccoNOQP*. The second FnrL binding site located within the *ccoN* gene may be used for regulation of downstream cytochrome biogenesis proteins *ccoGHIS* since FnrL represses this second downstream operon. To this point, it is likely that the actual protein content of assembled cytochrome *cbb*_*3*_ is lower even with higher RNA transcription levels of *ccoNOQP*.

**Fig. 5:**
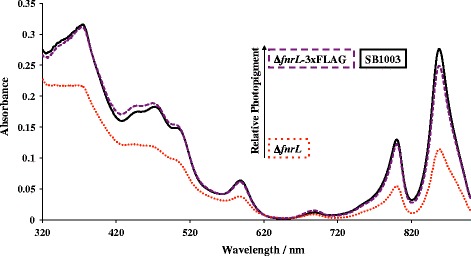
*fnrL* mutant in *Rba. capsulatus* show a reduction in photopigments. Photopigments from whole cell extracts of *Rba. capsulatus* shown in solid black, *fnrL* mutant in dotted red and *fnrL* mutant complemented with 3xFLAG tag shown in dashed purple

**Fig. 6:**
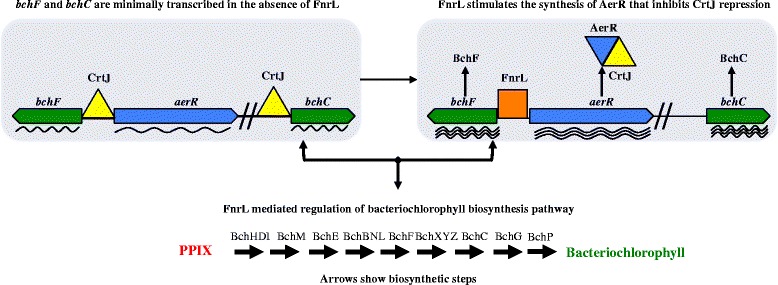
Schematic of Bacteriochlorophyll regulation. Repressor and antirepressor CrtJ/AerR system is shown as yellow/blue triangles, FnrL as an orange square, genes as chevrons and biosynthetic steps are shown as straight arrows. Wavy arrows indicate mRNA transcripts

Even though the Δ*fnrL* strain is capable of photosynthetic growth, it appears that FnrL is directly involved in regulating photosynthesis in this species. This conclusion is supported by spectral analysis of anaerobically grown Δ*fnrL* mutant strain of *Rba. capsulatus* which exhibits a clear reduction in photosystem spectral components relative to that observed with wild type cells (Fig. 5). A mechanism for this reduction in pigment synthesis is revealed by the presence of an FnrL ChIP-seq peak containing a FnrL recognition sequence in the intergenic region between the divergently transcribed bacteriochlorophyll biosynthesis gene *bchF* and the bacteriochlorophyll regulator *aerR* (Fig. 4d). Two potential FnrL binding sites were identified within the *bchF-aerR* intergenic region with both sites exhibiting good similarity to the consensus sequence. AerR is a cobalamin binding anti-repressor of the bacteriochlorophyll/carotenoid/light harvesting repressor CrtJ and thus the 2-fold activation of AerR expression by FnrL would relieve repression by CrtJ (Fig. 6) [1]. Furthermore this RNA–seq data is validated by a previous *in vivo* expression study using *lacZ* reporter plasmids which showed that AerR expression increases 2-fold under anaerobic conditions [7, 23].

We have also identified FnrL binding sites in the *puc* and *puf* light harvesting and reaction center operons (Additional file 1: Table S1). Specifically, there is a FnrL site that overlaps with the translational start site of *pucA* as well as a second site located 250 bp downstream of the start codon of *pucC*. The expression of *pucB* and *pucDE* up-regulated by FnrL indicating one or both of these sites may indeed be involved in activation of *puc* operon expression. There is also a ChIP-seq peak that spans the genetic space of *pufLM* with an FnrL binding sequence within *pufM* (42 bp upstream of the *pufX* start codon). RNA-sequencing show that *pufLM* is also up-regulated.

### FnrL has a limited but suppressing role in motility

A number of flagellar, chemotaxis, aerotaxis and gas vesicle genes are either directly or indirectly repressed by FnrL (Additional files 1 and 2: Table S1 and S2). Many structural flagellar genes are located, in large part, in five operons. RNA-seq and ChIP-seq results indicate that FnrL directly represses a 5-gene operon (*rcc03522*- *rcc03525*) that codes for an unknown function flagellar protein, FlbT, FlaF, and FlaA (flagellin protein needed for synthesis of the flagella filament). A ChIP-seq peak was observed that spans this operon with a consensus FnrL binding site located 42 bp upstream of the FlbT start codon (Table 1).

In addition to flagellar structural proteins, FnrL also represses *cheA1* that codes for chemotaxis signal transduction protein, a number of methyl-accepting chemotaxis receptors (*rcc00644*, *rcc02611 rcc02887, rcc02139*, and *rcc01667*), two aerotaxis receptors (*rcc02075* and *rcc03176*) and several gas vesicle proteins (*rcc01054* and *rcc01056*) (Table 1, Additional files 1 and 2: Table S1, and S2). One possible explanation for FnrL repression of motility may be that there is selective pressure to suppress motility under anaerobic photosynthetic growth conditions where light driven energy production is not limiting. Under photosynthetic growth conditions these metabolically diverse cells are very capable of directly synthesizing all essential cellular metabolites and likely not as reliant on chemotaxis. Repression of these motility components by FnrL would be relieved in the presence of oxygen that would disrupt the DNA binding activity of FnrL. This would allow the cell to synthesize components needed to either aerotax to areas with increasing oxygen content or increase their buoyancy so that they can rapidly “float” in an aquatic environment towards an oxygen source.

### FnrL’s role in anaerobic carbon metabolism

**Fig. 7:**
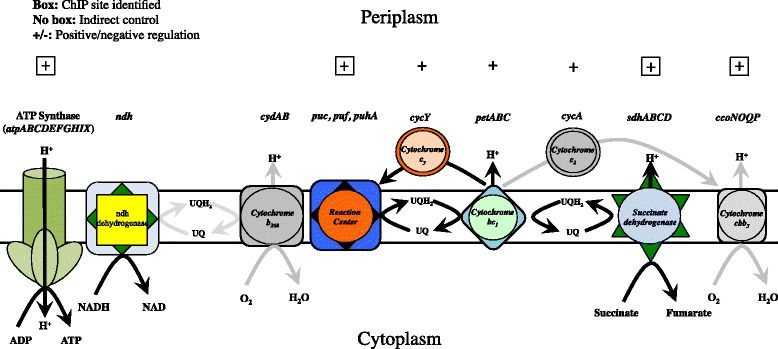
Schematic of *Rba. capsulatus* respiration. Boxes represent direct control by FnrL and activation/repression is shown as +/−

FnrL is not directly involved in glycolysis or gluconeogenesis; however, there are two of steps in glycolysis/gluconeogenesis that are indirectly activated such as phosphopyruvate hydratase (*rcc01715*) and glyceraldehyde-3-phosphate dehydrogenases (rcc02160). However, tricarboxylic acid cycle (TCA) genes are directly activated by FnrL (also called the Krebs cycle and the citric acid cycle) (Additional file 2: Table S2). Of the TCA genes, succinate dehydrogenase is directly activated by FnrL and contains a consensus binding sequence 26 bp upstream of the *sdhD* start codon and within *sdhC* coding region. Succinate dehydrogenase in turn provides reducing power to ubiquinone to drive cytochrome *bc*_*1*_ (*petABC*) complex that is indirectly activated (Fig. 7).

*Rba. capsulatus* contains two forms of RuBisCO where form I is coded by *cbbLS* and form II is coded by *cbbM*. Form I and II *cbb* operons are regulated by related LysR family transcription factors CbbR_I_ and CbbR_II_, respectively. FnrL does not control these regulators, but deletion of *fnrL* causes the, expression of *cbbLS* to be reduced.

### Regulation of tetrapyrrole biosynthesis and iron transport by FnrL

The common trunk of the tetrapyrrole pathway from δ-aminolevulinic acid to uroporphyrinogen III is used for cobalamin, heme and bacteriochlorophyll biosynthesis [5, 7, 24]. There is indirect activation of *hemA* expression (Additional file 2: Table S2) with possible direct activation of ferrochelatase (*hemH*) expression with a predicted FnrL binding site that shows good similarity to the FnrL consensus recognition sequence. While there is no detectable FnrL binding site in the intergenic region between divergently transcribed *hemB* and *rcc01809* genes, there is a ChIP-seq peak with an FnrL recognition sequence located within *rcc01809*. This suggests that the promoter for *hemB* may be within the rcc01809 coding sequence. Interestingly, FnrL has an indirect role in repressing cobalamin (*cob* gene) synthesis (Additional file 2: Table S2). We hypothesize that the cell attenuates cobalamin biosynthesis in order to divert intermediates for the biosynthesis of PPIX and bacteriochlorophyll (unpublished observation).

We did not find any direct regulation of FnrL on siderophore or iron transport genes. Iron is an essential component of heme as well as the redox responding cofactor in FnrL and we were surprised to find a limited direct role of FnrL in iron transport. We did observe that FnrL does indirectly repress a siderophore ABC transporter (*rcc02116*), a FeoA family protein (*rcc02028*), a Fe(III) type ABC transporter (*rcc02579*) and FeoA2 that codes for a ferrous iron transporter (*rcc00091*) (Additional file 2: Table S2). One of the highest enriched (21-fold) sites was found in one uncharacterized set of genes (*rcc3401*-*rcc3402*) the first of which is a band 7/SPFH family protein thought to be the core of an ion channel while the second is a hypothetical protein that shares 24 % identity to a membrane protease found to be important for virulence in *P. gingivalis* W83 [25]. These two genes are typically found in an operon and appear to form the foundation of an ion channel. The role of this gene cluster is unclear in *Rba. capsulatus,* but it may be used for acquiring or sensing depleting ions including iron. Indeed it has been found that a knockout of homologous gene cluster in *S. oneidensis* shows a strong effect on iron metabolism with the disruption leading to a decrease in intracellular iron which affected proteins involved in respiratory chain that utilize iron [26].

### Comparison of FNR/FnrL differentially expressed genes in *Rba. capsulatus*, *Rba. sphaeroides*, and *E. coli*

The number of genes that encompass the *Rba. capsulatus* FnrL regulon (807 genes) is similar to the number of genes reported for the *Rba. sphaeroides* FnrL regulon (917 genes) [6, 18]. However, analysis for congruence shows that only 171 genes are differentially expressed in common (Tables 2 and 3 in Additional file 6: Table S6). This means that 78 and 81 % of the genes in the *Rba. capsulatus* and *Rba. sphaeroides* FnrL regulons, respectively, are uniquely regulated by FnrL in these photosynthetic species [18]. Among the 171 commonly regulated genes, 52 are convergently activated and 36 are convergently repressed with 83 exhibiting differences in regards to activation versus repression. Divergent roles of FnrL in these species is also highlighted by the fact that only 9 FnrL ChIP-seq peaks are located in common positions relative to a common downstream gene out of the 82 FnrL peaks in *Rba. capsulatus* and 28 FnrL peaks in *Rba. sphaeroides* (Additional file 7: Table S7).

**Table 2:** Comparison of selected genes directly controlled by FnrL in *Rba. capsulatus* and *Rba. sphaeroides*

Locus ID^a^	Locus ID^b^	Gene Name	Function	Regulation^c^	FnrL Recognition Sequence
*Unique to Rhodobacter sphaeroides*					
RSP0820	RCC00426	*cytochrome b*_*561*_	electron transporting and shutting	+	TTGATGCGGATCAA
RSP2984	RCC00147	*hemA*	5-aminolevulinate synthase	+	TTGATAAGGATCAA
RSP0317	RCC00151	*hemN*	Coproporphyrinogen III oxidase	+	TTGCGCAGGATCAA
RSP1819	RCC00091	*feoA*	ferrous iron transport protein	+	TTGACGCGGATCAA
RSP1949	RCC01601	*Iron/Sulfur*	FeS assembly SUF system protein	+	GTGATCTGCATCAA
RSP0100	RCC01517	*nuoA*	NADH dehydrogenase	+	CTGATGCAGATCAA
*Unique to Rhodobacter capsulatus*					
RSP0278	RCC02532	*pucC*	light harvesting protein	+	CTGATCGGCTTCAA
RSP0284	RCC00666	*bchF*	2-vinyl bacteriochlorophyllide hydratase	+	ATGACATGGGTCAA
RSP0283	RCC00667	*aerR/ppaA*	regulatory protein PpaA	+	ATGCTCGAGTTCAA
RSP1149	RCC01729	*oxidoreductase*	oxidoreductase	+	ATGATCCAAGTCAT
*Directly activated in both organisms*					
RSP0775	RCC02479	cytochrome *c*	peroxidase	+	CTGATGCAGCGCAA
			RSP Recognition sequence		TTGACGCAGATCAG
RSP0696	RCC01157	*ccoN*	cbb3-type cytochrome c oxidase subunit I	+	TTGATCAAGGTCAA
			RSP Recognition sequence		TTGATCCTCATCAA
RSP3044	RCC02849	*dorS*	DMSO/TMAO-sensor hybrid histidine kinase	+	TTGATCGGGATCAA
			RSP Recognition sequence		TTGACGTCAATCAA
RSP0166	RCC02590	*dksA2*	DnaK suppressor protein	+	TTGATTCAGGTCAA
				RSP Recognition sequence	TTGATGCAGGTCAA
RSP2247	RCC01495	*fusA*	translation elongation factor G	+	TTGGCATGGGTCAA
			RSP Recognition sequence		TTGATTCAGGTCAA
RSP0697	RCC01156	*uspA*	universal heath shock protein	+	TTGACGCGGATCAA
			RSP Recognition sequence		TTGATCCATGTCAA
RSP2337	RCC01723	*ccpA1*	cytochrome-c peroxidase	+	TTGATCTGCGTCAA
			RSP Recognition sequence		TTGATCTGCGTCAT
RSP0698	RCC02493	*fnrL*	CRP/FNR family transcriptional regulator	+	TTGTCCCAAATCAA
			RSP Recognition sequence		TTGATTCAGATCAA
RSP0467	RCC01733	*ubiD*	3-octaprenyl-4-hydroxybenzoate carboxy-lyase	N.D.	TTGATCAATATCAA
			RSP Recognition sequence		TTGATGTAGGTCAA

N.D No differential gene expression based on RNA-seq (Not Determined)

RSP Recognition sequence *Rba. sphaeroides* recognition sequence

^a^ *Rba. sphaeroides*

^b^ *Rba. capsulatus*

^c^(+/-) indicate activation/repression by FnrL

The large number of uniquely regulated genes in these two *Rhodobacter* species indicates that FnrL has adopted dissimilar regulatory roles. This conclusion is highlighted by divergent roles of FnrL in regards to the regulation of tetrapyrrole biosynthesis and photosystems. For example, FnrL directly activates *hemA* in *Rba. sphaeroides* but not in *Rba. capsulatus.* Bacteriochlorophyll genes *bchM, bchJ, bchO, and bchD* are also convergently repressed by both species while *bchC*, *bchE* and *bchF* are activated in *Rba. capsulatus* and repressed in *Rba. sphaeroides.* Furthermore, an FnrL ChIP signal is observed in the light harvesting complex *pufALM* operon from *Rba. capsulatus* which is positively regulated by FnrL, but not in *Rba. sphaeroides* where this operon appears to be negatively regulated by FnrL [18]*.* This difference also extends to downstream secondary photosystem regulators. Specifically, we found an FnrL ChIP signal in the *Rba. capsulatus* promoter region of AerR which is a photosystem regulator that functions as an antirepressor of the *bch*/*crt* repressor CrtJ [1–5]. In *Rba. sphaeroides* the control of this downstream regulator by FnrL does not appear to exist [18]*.* These differences signal that there is significant variation in the role of FnrL for the control of photosystem synthesis between these species.

Some notable similarities do, however, exist between these *Rhodobacter* species. For example, FnrL directly ctivates DMSO reductase and *cbb*_*3*_ cytochrome oxidases and has direct negative effects on cobalamin biosynthesis in both of these species (Table 2). Furthermore, both organisms use FnrL to indirectly activate *cbbLS* (Calvin-Benson-Bassham cycle).

Searching for convergence of FnrL/FNR regulons across genera we observed that there is only a handful of examples where the *E. coli* FNR regulon shows congruence with either of the *Rhodobacter* regulons. For example, the DMSO reductase system and *uspA* (universal stress protein) is directly activated by FnrL/FNR in all three species (Additional files 8, 9 and 10: Tables S8, S9, S10) [6]. Similarly, the *fadBA* (fatty acid metabolism) operon is repressed in all three species though in all cases this repression appears to be indirect. The *E. coli* and *Rba. capsulatus* FNR/FnrL orthologues also directly control *nrdD* (anaerobic ribonucleoside reductase) but this does not appear to be the case in *Rba. sphaeroides*. These results clearly demonstrate that there exist considerable divergence in function of FNR/FnrL orthologues from distant and more closely related bacteria.

## Conclusions

**Fig. 8:**
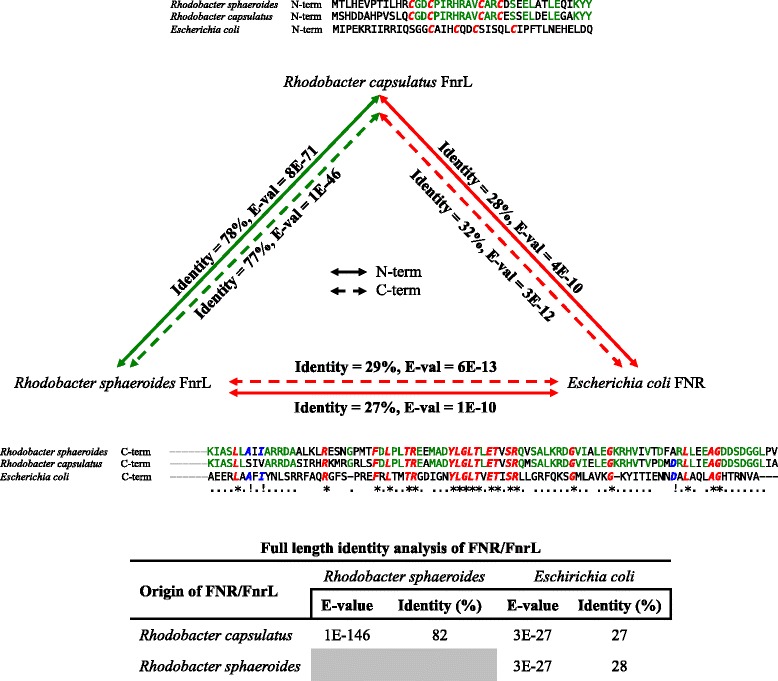
Comparison of FNR/FnrL homologues. Similarities of FnrL from *Rba. capsulatus* and *Rba. sphaeroides* and their differences to FNR from *E. coli.* Fe/S motif was taken from the N-terminus (solid) and HTH domain was taken from the C-terminus (dashed)*.* Red colored amino acids denote critical residues, green denote similarities between *Rhodobacter* species, blue denote similarities between *E. coli* and *Rhodobacter* species, grey are unique to each organism and redundantly represented by ‘.’, ‘*’ and ‘!’

**Fig. 9:**
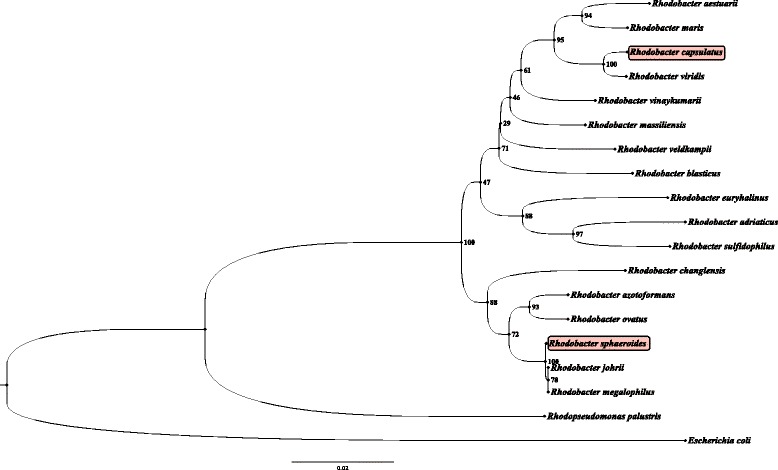
Phylogenetic relatedness of Rhodobacter species. The evolutionary history was inferred using the Neighbor-Joining method [42]. The bootstrap consensus tree inferred from 1000 replicates [43] is taken to represent the evolutionary history of the taxa analyzed [43]. The evolutionary distances were computed using the Maximum Composite Likelihood method [44] and are in the units of the number of base substitutions per site. The analysis involved 19 16S rRNA sequences. All positions containing gaps and missing data were eliminated. There were a total of 1325 positions in the final dataset. Evolutionary analyses were conducted in MEGA6 [45].

This study shows that genes constituting the FnrL regulon from *Rba. capsulatus* are remarkably dissimilar from the published FnrL regulon from *Rba. sphaeroides.* Indeed only 9 genes in these two photosynthetic species have FnrL binding sites upstream from common targets. This dissimilarity is striking given that these organisms share similar anoxygenic photosynthetic physiologies and therefore presumably face similar challenges in controlling energy balance (redox poise) in response to light, oxygen, and nutrient availability. The fact that these FnrL orthologues exhibit high sequence identity (Fig. 8) and utilize similar target sequences (Fig. 2), and yet control many different target genes, indicates that there is significant evolutionary drift in the location of transcription factor recognition sequences even among related species that occupy similar environmental niches (Fig. 9).

It is informative to note similarities and differences that exist between these *Rhodobacter* FnrL regulons as this can highlight areas of conservation that may apply to a broad spectrum of alpha-proteobacteria. For example, iron transport is controlled by FnrL in *Rba. sphaeroides* but not in *Rba. capsulatus* (Table 2) [27, 28]. Differences also exist for heme synthesis where FnrL from *Rba. sphaeroides* directly controls *hemA*, *hemN* and *hemZ* while FnrL in *Rba. capsulatus* is not directly involved in heme biosynthesis with the possible exception of *hemH*. We also note that numerous cobalamin biosynthesis genes are indirectly down-regulated by FnrL in both *Rhodobacter* species. This may not be an intuitive result since cobalamin is needed for anaerobic biosynthesis of bacteriochlorophyll where BchE uses cobalamin as its cofactor [29]. However, both *Rhodobacter* species undergo an extensive increase in bacteriochlorophyll biosynthesis (>100-fold) when they are grown anaerobically and yet both species show FnrL mediated repression of the cobalamin pathway.

In regards to the FNR regulon from *E. coli* [6]*,* this species does not possess the ability to undergo photosynthesis and anaerobically relies on fermentative growth. Consequently, member of the *E. coli* FNR regulon are quite divergent from that of the *Rba. capsulatus* and *Rba. sphaeroides* FnrL regulons. Indeed despite the large number of genes that constitute the FNR/FnrL regulons from these species, we only found a few instances where all three organisms have direct orthologues that share the same direct FNR/FnrL control; the DMSO reductase system and the universal stress protein *uspA.* Although all three species do not share direct cytochrome oxidase orthologues, all three organisms do use FnrL/FNR to control the expression of oxygen utilizing terminal respiratory chain components [13, 16, 30, 31].

Finally, an example of metabolic divergence of *E. coli* from *Rhodobacter* species is highlighted by the direct involvement of *E. coli* FNR in regulating glycolysis while in the *Rhodobacter* species FnrL is not directly involved. Logically, in a non-photosynthetic organism such as *E. coli* it makes sense to direct phosphoenolpyruvate for either aerobic or anaerobic growth by an oxygen sensing transcriptional factor while it appears that both *Rhodobacter* species have adopted alternate modes of glycolytic routing mechanisms [6]. FnrL’s from *Rba. capsulatus* and *Rba. sphaeroides* are also indirectly involved in cobalamin repression while *E. coli* does not undertake *de novo* cobalamin biosynthesis and instead must go through a cobinamide intermediate [32].

The divergences observed with the FnrL/FNR regulons from *Rba. capsulatus, Rba. sphaeroides* and *E. coli* highlights the fact that analysis of transcription factor regulons must be experimentally derived on an individual basis as corollary regulatory events clearly differ between closely related organisms. This divergence can occur even among highly homologous transcription factor orthologs that bind to similar recognition sequences.

## Methods

### Strains, media, and growth conditions

The *Rba. capsulatus* parental strain SB1003, and its Δ*fnrL* derivative have previously been described [16]. These strains were routinely grown in peptone/yeast extract (PY) either in liquid or on agar plates with liquid media supplemented with MgCl_2_ and MgSO_4_ to a final concentration of 2 mM. Biological replicate strains were first grown semi-aerobically overnight as a 5 ml PY culture in culture tubes at 34 °C shaking at 200 rpm. Subsequently, these cultures were transferred and grown anaerobically in screw-cap vials overnight at 34 °C with four 75 W light bulbs after which the cells were subcultured to an optical density of 0.03 and spectrally monitored until harvesting at OD_660_ ~ 0.3. The optical density in the anaerobic vials was checked using Unico 1100 RS Spectrophotometer.

### RNA isolation, validation, and sequencing (RNA-Seq)

After cultures reached OD_660_ ~ 0.3 the cultures were harvested by placing immediately into an ice/water bath and then transferred into 2 mL Eppendorf tubes, centrifuged at 6000 rpm for 3 min at 4 °C. The entire 2 mL cell pellet was then used for extracting total RNA using a Bioline Isolate II RNA extraction kit. Briefly, the bacterial pellet was dissolved in 100 μL of TE (10 mM Tris–HCl, 1 mM EDTA, pH 8) buffer containing 10 mg/mL lysozyme and incubated for 3 min at room temperature. After isolation of total RNA the DNA was removed by addition of 1 unit of Turbo DNAse and further incubated for 30 min at 37 °C. A cleanup step was performed with Zymogen Direct-zol RNA extraction kit according to manufacturers instructions. To check for residual DNA, qRT-PCR of the *rpoZ* housekeeping gene was performed with and without reverse transcriptase.

Total RNA was submitted to the University of Wisconsin-Madison Biotechnology Center where it was verified for purity and integrity with a NanoDrop2000 Spectrophotometer and Agilent 2100 BioAnalyzer, respectively. Samples that met Illumina sample input guidelines were prepared according the TruSeq® Stranded Total RNA Sample Preparation Guide (15031048 E) using the Illumina TruSeq® Stranded Total RNA kit (Illumina Inc., San Diego, California, USA) with minor modifications. For each library preparation, 2 μg of total RNA was reduced of ribosomal RNA using the EpiCentre RiboZero™ rRNA Removal (Bacteria) kit (EpiCentre Inc., Madison, WI, USA) as directed. Subsequently, each rRNA-depleted sample was fragmented using divalent cations under elevated temperature. The fragmented RNA was synthesized into first strand cDNA using SuperScript II Reverse Transcriptase (Invitrogen, Carlsbad, California, USA) combined with Actinomycin D and random primers followed by second strand synthesis using Second Strand Marking Master Mix. The blunt-ended double-stranded cDNA was purified by paramagnetic beads (Agencourt AMPure XP beads (Beckman Coulter, Indianapolis IN, USA). The cDNA products were incubated with A-Tailing Mix to add an ‘A’ base (Adenine) to the 3′ end of the blunt DNA fragments followed by ligation to Illumina adapters, which have a single ‘T’ base (Thymine) overhang at their 3′end. The adapter-ligated products were purified by paramagnetic beads. Adapter ligated DNA was then amplified in a Linker Mediated PCR reaction (LM-PCR) for 10 cycles using the PCR Master Mix and PCR Primer Cocktail and purified by paramagnetic beads. Quality and quantity of the finished libraries were assessed using an Agilent DNA1000 chip (Agilent Technologies, Inc., Santa Clara, CA, USA) and Qubit® dsDNA HS Assay Kit (Invitrogen, Carlsbad, California, USA), respectively and standardized to 2 μM. Cluster generation was performed using standard Cluster Kits (v3) and the Illumina Cluster Station. Single 100 bp sequencing was performed, using standard SBS chemistry (v3) on an Illumina HiSeq2000 sequencer. Images were analyzed using the standard Illumina Pipeline, version 1.8.2.

### Construction and sequencing of ChIP libraries (ChIP-Seq)

A plasmid expressing a FnrL 3xFLAG Tag with an isopropyl β-D-1-thiogalactopyranoside (IPTG) inducible lac promoter was constructed with the following reverse primer ctaGCTAGCttaCTTGTCATCGTCATCCTTGTAGTCGATGTCATGATCTTTATAATCACCGTCATGGTCTTTGTAGTCggatc containing NheI restricted site and forward primer acatGCATGCGGTTCATCCCCGATTGCGCCAG containing SphI restriction site and cloned into pSRK (complementation plasmid containing gentamycin resistance marker to produce pSRK-FnrL. This expression plasmid is described in detail in the following reference [33]. pSRK-FnrL was subsequently mated into *Rba. capsulatus* using S17-1 *E. coli* mating strain with complementation checked by growing cells anaerobically with 50 mM DMSO in the presence of 1.0 mM IPTG. FnrL mutants fail to utilize DMSO as a terminal electron acceptor due to their inability to express sufficient amounts of DMSO reductase [16] and also have reduced levels of photopigments (Fig. 5). The FnrL deletion strain complemented with pSRK-FnrL was subsequently able to restore growth on DMSO and to resort wild type photopigment levels (Fig. 5) identical to that of wild type cells.

Photosynthetically grown FnrL-3xFLAG complemented cells were treated with 37 % formaldehyde to a final concentration of 1 % for 15 min at room temperature. Crosslinking with formaldehyde quenched by the addition of Tris–HCl pH 8.2 to a final concentration of 500 mM for 5 min at room temperature after which the cells were harvested by centrifugation. The cells were washed with 40 mL TBS buffer and resuspended in 4 mL buffer composed of 50 mM Tris pH 7.5, 150 mM NaCl, 1 mM EDTA, 1 % Triton X100. After disruption by French press lysis, the DNA was sheared three times by sonication using a small tip sonicator with 15-W power output. Protein bound to DNA was then reverse crosslinked by heating to 65 °C overnight with concurrent removal of contaminating RNA by the addition of 1 μg of RNAse A per 100 μL sample. Immunoprecipitation was performed according to manufacturers instruction using ANTI-FALG® M2 Affinity Gel (Cat. Number A2220).

Purified immunoprecipitated and input DNA was submitted to the University of Wisconsin-Madison Biotechnology Center for library construction and sequence analysis. DNA concentration and sizing were verified using the Qubit® dsDNA HS Assay Kit (Invitrogen, Carlsbad, California, USA) and Agilent DNAHS chip (Agilent Technologies, Inc., Santa Clara, CA, USA), respectively. Samples that met the Illumina sample input guidelines were prepared according the TruSeq® ChIP Sample Preparation kit (Illumina Inc., San Diego, California, USA) with minor modifications. Libraries were size selected for an average size of 350 bp using SPRI-based bead selection. Quality and quantity of the finished libraries were assessed using an Agilent DNA1000 chip and Qubit® dsDNA HS Assay Kit, respectively with DNA concentration standardized to 2 μM. Cluster generation was performed using standard Cluster Kits (v3) and the Illumina Cluster Station. Single 100 bp sequencing was performed, using standard SBS chemistry (v3) on an Illumina HiSeq2000 sequencer. Images were analyzed using the standard Illumina Pipeline, version 1.8.2.

### Data pre-processing, computer software and data analysis for RNA-sequencing and ChIP-sequencing

All computations were performed on a custom built computer running Ubuntu 13.10 equipped with Asus Z9PE-D8 WS motherboard, 2 x Intel Xeon E5-2630 V2 CPU, 128 GB DDR3-1600 RAM. Each fastq file was checked for good quality using FastQC and trimmed of low quality sequences using Trimmomatic program using a sliding window of 5:25 and a minimum length of 40. The reads were aligned to the genome using Bowtie2 [34] mapped individual genes using HTSeq-count [35]. Raw counts generated from HTSeq-count program were used to generate differentially expressed genes with DESeq2 package in R [36, 37]. Default parameters with noted exceptions were used for Trimmomatic, Bowtie2 and HTSeq-count programs.

For processing ChIP-seq, a pipeline consisting of Trimmomatic with a sliding window of 5:25 and a minimum length of 40 was used to trim poor quality reads, Bowtie2 to align the reads to the SB1003 reference genome, MACS to determine significantly enriched sites, and MEME for binding sequence extraction using default parameters [38]. All packages are available for download via github and/or bioconductor [33–35, 38–40]. Raw sequence data from our RNA-seq and ChIP-seq analysis can be accessed via NCBI Sequence Read Archive server under the accession number (PRJNA274121).

### Cross-species orthologous analysis

Orthologues of *Rba. capsulatus* in *Rba. sphaeroides* and *E. coli* were found using OMA web server accessible at http://http://www.omabrowser.org [41]. Data sets for *Rba. sphaeroides* and *E. coli* used for differential gene expression comparison were taken directly from published results and presented in congruent style [6, 18].

### Spectral scans of SB1003 and FnrL

Wild-type *Rba. capsulatus*, *fnrL* mutant and *fnrL* mutant complemented with 3xFLAG tag were grown in PY medium until OD_660 nm_ reached 0.3 and 2 mL of each genotype was harvested and centrifuged to collect the pellet. The pellet was dissolved in buffer containing 20 mM Tris and 150 mM NaCl and sonicated three times using power of 9 W for duration of 15 s. The samples were clarified by centrifugation and the spectra were recorded.

## Additional files

Additional file 1: Table S1. Genes containing ChIP-seq signal with corresponding RNA-seq expression. (XLSX 40 kb) Additional file 2: Table S2. A table of genes indirectly controlled by FnrL in *Rba. capsulatus*. (DOCX 228 kb) Additional file 3: Table S3. FnrL ChIP-seq signal with corresponding *p*-value > 0.05 based on RNA-seq expression change. (DOCX 66 kb) Additional file 4: Table S4. FnrL predicted binding site based on PWM with corresponding RNA-seq expression change. (DOCX 165 kb) Additional file 5: Table S5. Clusters of orthologous groups definitions. (DOCX 66 kb) Additional file 6: Table S6. Orthologues of *Rba. capsulatus* in *Rba. sphaeroides* and their differential expression. (XLSX 280 kb) Additional file 7: Table S7. Orthologues of *Rba. capsulatus* in *Rba. sphaeroides* and presence of FnrL ChIP signals. (XLSX 67 kb) Additional file 8: Table S8. Orthologues of *Rba. capsulatus* in *E. coli* and their differential expression. (XLSX 85 kb) Additional file 9: Table S9. Orthologues of *Rba. capsulatus* in *E. coli* and presence of FnrL/FNR ChIP signals. (XLSX 57 kb) Additional file 10: Table S10. Orthologues of *Rba.spaeroides* in *E. coli* and their differential expression. (XLSX 63 kb)
