# Supplementary material for: Analysis of the FnrL regulon in Rhodobacter capsulatus reveals limited regulon overlap with orthologues from Rhodobacter sphaeroides and Escherichia coli
Source: BMC Genomics. 2015 Nov 4;16:895. doi: 10.1186/s12864-015-2162-4 (PMC4634722; doi:10.1186/s12864-015-2162-4)
Supplement: Additional file 2: Table S2. — A table of genes indirectly controlled by FnrL in Rba. capsulatus. (DOCX 228 kb) [file 12864_2015_2162_MOESM2_ESM.docx]

| **Table S2:** Genes indirectly controlled by FnrL | | |  |  |
| --- | --- | --- | --- | --- |
| **Locus ID** | **Gene** | **Description** | **Fold Change** | **p-val** |
| **COG C: Energy production and conversion** | | | | |
| RCC02845 | *torA* | trimethylamine-N-oxide reductase | 3 | 0 |
| RCC02847 | *torC* | trimethylamine-N-oxide reductase c-type cytochrome | 2.87 | 0 |
| RCC01728 | *nifJ* | pyruvate-flavodoxin oxidoreductase | 2.26 | 0 |
| RCC03284 | *fdxN* | ferredoxin I | 2.06 | 0.003 |
| RCC02791 | *fdxA* | ferredoxin II | 1.89 | 0 |
| RCC02501 | *cycY* | cytochrome c | 1.85 | 0.001 |
| RCC03285 | *fdxC* | ferredoxin IV | 1.84 | 0.027 |
| RCC00889 | *NosL family protein* | NosL family protein | 1.72 | 0.045 |
| RCC02681 | *cytochrome b561* | cytochrome b561 family protein | 1.71 | 0.009 |
| RCC00436 | *cytB* | cytochrome B561 | 1.64 | 0 |
| RCC02682 | *cytochrome c'* | cytochrome c' | 1.59 | 0.003 |
| RCC00736 | *sdhB* | succinate dehydrogenase, iron-sulfur subunit | 1.58 | 0.002 |
| RCC00588 | *anfK* | nitrogenase iron-iron protein subunit beta | 1.54 | 0.044 |
| RCC03258 | *cytochrome c biogenesis* | cytochrome c biogenesis protein transmembrane region | 1.53 | 0.014 |
| RCC00720 | *sucC* | succinyl-CoA synthetase (ADP-forming) subunit beta | 1.48 | 0.012 |
| RCC02974 | *atpH* | ATP synthase F1 subunit delta | 1.47 | 0.024 |
| RCC00740 | *atpI* | ATP synthase F0 subunit I | 1.45 | 0.008 |
| RCC00578 | *cbbS* | ribulose bisphosphate carboxylase small subunit | 1.44 | 0.023 |
| RCC01705 | *pdhA* | pyruvate dehydrogenase | 1.43 | 0.019 |
| RCC02448 | *aldH2* | aldehyde dehydrogenase | 1.43 | 0.011 |
| RCC01887 | *icd* | isocitrate dehydrogenase | 1.42 | 0.042 |
| RCC01517 | *nuoA* | NADH-quinone oxidoreductase subunit A | 1.41 | 0.03 |
| RCC03048 | *lldD* | L-lactate dehydrogenase | 1.41 | 0.002 |
| RCC00516 | *tpl* | tyrosine phenol-lyase | 1.4 | 0.007 |
| RCC03243 | *ccrA* | crotonyl-CoA reductase | 1.4 | 0.031 |
| RCC02826 | *fumC* | fumarate hydratase | 1.4 | 0.038 |
| RCC00832 | *pta* | phosphate acetyltransferase | 1.38 | 0.006 |
| RCC00724 | *sucA* | oxoglutarate dehydrogenase | 1.37 | 0.022 |
| RCC01203 | *pyc* | pyruvate carboxylase | 1.35 | 0.036 |
| RCC02769 | *petB* | ubiquinol--cytochrome-c reductase | 1.32 | 0.046 |
| RCC01240 | *cycA1* | cytochrome c2 | 1.32 | 0.047 |
| RCC01378 | *aldehyde dehydrogenase* | aldehyde dehydrogenase | 1.3 | 0.05 |
| RCC02837 | *pckA* | phosphoenolpyruvate carboxykinase | 1.3 | 0.042 |
| RCC02871 | *glcD* | glycolate dehydrogenase, subunit GlcD | 1.29 | 0.035 |
| RCC01817 | *bdhA* | 3-hydroxybutyrate dehydrogenase | 1.29 | 0.031 |
| RCC00524 | *gyaR1* | glyoxylate reductase | 1.28 | 0.033 |
| RCC03496 | *maeB2* | malate dehydrogenase | 1.27 | 0.039 |
| RCC00413 | *gabD* | succinate-semialdehyde dehydrogenase | 1.25 | 0.043 |
| RCC00368 | *aroH* | hydroxyphenylpyruvate synthase | -1.31 | 0.047 |
| RCC00577 | *cbbQ* | CbbQ protein | -1.31 | 0.029 |
| RCC00764 | *hupU* | uptake hydrogenase small subunit | -1.46 | 0.044 |
| RCC02015 | *aldH1* | aldehyde dehydrogenase | -1.47 | 0.015 |
| RCC03036 | *fdhB* | NAD-dependent formate dehydrogenase subunit beta | -1.48 | 0.015 |
| RCC01608 | *cytochrome c oxidase assembly* | cytochrome c oxidase assembly protein subunit 15 | -1.53 | 0.007 |
| RCC00592 | *hyi* | hydroxypyruvate isomerase | -1.53 | 0.029 |
| RCC02970 | *atpC* | ATP synthase F1 subunit epsilon | -1.56 | 0.009 |
| RCC00798 | *dehydrogenase* | D-isomer specific 2-hydroxyacid dehydrogenase | -1.59 | 0.002 |
| RCC01862 | *xerD* | tyrosine recombinase XerD | -1.6 | 0.006 |
| RCC03439 | *fadH* | 2,4-dienoyl-CoA reductase | -1.61 | 0.004 |
| RCC03253 | *cydC* | cysteine ABC transporter ATP-binding protein/permease | -1.64 | 0.03 |
| RCC01742 | *mcr* | alpha-methylacyl-CoA racemase | -1.64 | 0.009 |
| RCC03037 | *fdhC* | NAD-dependent formate dehydrogenase subunit gamma | -1.74 | 0.002 |
| RCC00817 | *ldh* | L-lactate dehydrogenase | -1.76 | 0.008 |
| RCC03373 | *ackA3* | acetate kinase | -1.76 | 0.009 |
| RCC01480 | *transmembrane pair family* | transmembrane pair family protein | -1.84 | 0.001 |
| RCC00761 | *hoxH* | NAD-reducing hydrogenase HoxS subunit beta | -2.24 | 0 |
|  | | |  |  |
| **COG D: Cell cycle control, cell division, chromosome partitioning** | | |  |  |
| RCC03192 | *ftsH* | cell division protease FtsH | 1.38 | 0.002 |
| RCC02388 | *ispZ* | intracellular septation protein A | 1.37 | 0.033 |
| RCC03193 | *tilS* | tRNA(Ile)-lysidine synthase | -1.46 | 0.016 |
| RCC00818 | *ftsW* | cell division protein FtsW | -1.48 | 0.01 |
| RCC00824 | *ftsQ* | cell division protein FtsQ | -1.51 | 0.048 |
|  | | |  |  |
| **COG E: Amino acid transport and metabolism** | | |  |  |
| RCC02959 | *potD5* | polyamine ABC transporter periplasmic polyamine | 1.75 | 0.001 |
| RCC01214 | *ilvC* | ketol-acid reductoisomerase | 1.67 | 0 |
| RCC03387 | *glnB2* | nitrogen regulatory protein P-II | 1.65 | 0.002 |
| RCC02765 | *ilvE1* | branched-chain-amino-acid transaminase | 1.62 | 0.003 |
| RCC00101 | *ABC transporter permease* | peptide/nickel transport system permease protein | 1.61 | 0.011 |
| RCC03161 | *dat* | D-amino-acid transaminase | 1.59 | 0 |
| RCC02711 | *asd* | aspartate-semialdehyde dehydrogenase | 1.56 | 0.01 |
| RCC01388 | *potD2* | polyamine ABC transporter periplasmic polyamine | 1.56 | 0.014 |
| RCC02357 | *trpG* | anthranilate synthase component II | 1.56 | 0.002 |
| RCC02183 | *potG2* | polyamine ABC transporter ATP-binding protein | 1.55 | 0.016 |
| RCC01226 | *urtB* | urea ABC transporter urea binding protein UrtB | 1.53 | 0.039 |
| RCC01183 | *hisB* | imidazoleglycerol-phosphate dehydratase | 1.52 | 0.001 |
| RCC01674 | *glnA3* | glutamine synthetase | 1.51 | 0.001 |
| RCC02777 | *glnA4* | glutamine synthetase-4 | 1.51 | 0.002 |
| RCC03408 | *hutG* | N-formylglutamate amidohydrolase | 1.51 | 0.016 |
| RCC03447 | *serA* | phosphoglycerate dehydrogenase | 1.51 | 0.007 |
| RCC03431 | *livK2* | branched-chain amino acid ABC transporter | 1.51 | 0.002 |
| RCC00338 | *bztD* | glutamate/aspartate ABC transporter ATP-binding protein | 1.49 | 0.019 |
| RCC00341 | *argB* | acetylglutamate kinase | 1.48 | 0.016 |
| RCC01816 | *family 5 extracellular solute-binding* | peptide/nickel transport system substrate-bind | 1.47 | 0.005 |
| RCC00895 | *proX1* | L-proline ABC transporter | 1.47 | 0.036 |
| RCC02268 | *potD3* | polyamine ABC transporter periplasmic polyamine | 1.46 | 0.019 |
| RCC01543 | *ilvI* | acetolactate synthase large subunit | 1.45 | 0.002 |
| RCC00438 | *glyA* | serine hydroxymethyltransferase | 1.44 | 0.014 |
| RCC00553 | *argC* | N-acetyl-gamma-glutamyl-phosphate reductase | 1.44 | 0.004 |
| RCC00082 | *leuD* | 3-isopropylmalate dehydratase small subunit | 1.43 | 0.007 |
| RCC00453 | *amino acid ABC transporter periplasmic* | polar amino acid transport | 1.42 | 0.001 |
| RCC03427 | *livG3* | branched-chain amino acid ABC transporter | 1.41 | 0.047 |
| RCC02175 | *mtbC* | dimethylamine corrinoid protein | 1.41 | 0.027 |
| RCC02801 | *pepF* | oligoendopeptidase F | 1.39 | 0.008 |
| RCC00354 | *pepT* | peptidase T | 1.38 | 0.005 |
| RCC01230 | *urtE* | urea ABC transporter ATP-binding protein UrtE | 1.37 | 0.043 |
| RCC01089 | *gsiB* | glutathione ABC transporter | 1.35 | 0.044 |
| RCC02182 | *class III aminotransferase* | K12256 putrescine aminotransferase | 1.35 | 0.01 |
| RCC02787 | *metB* | O-acetylhomoserine aminocarboxypropyltransferase | 1.34 | 0.02 |
| RCC03428 | *livH3* | branched-chain amino acid ABC transporter | 1.34 | 0.05 |
| RCC02774 | *hisJ* | polar amino acid ABC transporter | 1.33 | 0.032 |
| RCC03001 | *proA* | glutamate-5-semialdehyde dehydrogenase | 1.33 | 0.032 |
| RCC02780 | *glnA5* | glutamine synthetase-5 | 1.3 | 0.044 |
| RCC01129 | *trpB1* | tryptophan synthase subunit beta | 1.25 | 0.05 |
| RCC03473 | *gabT2* | 4-aminobutyrate aminotransferase | -1.33 | 0.029 |
| RCC02537 | *cysK1* | cysteine synthase | -1.41 | 0.05 |
| RCC02802 | *membrane dipeptidase* | Predicted Function | -1.46 | 0.009 |
| RCC02313 | *soxG* | sarcosine oxidase subunit gamma | -1.47 | 0.013 |
| RCC02359 | *trpC* | indole-3-glycerol phosphate synthase | -1.52 | 0.005 |
| RCC03472 | *thrB* | homoserine kinase | -1.57 | 0.006 |
| RCC02394 | *sdaA* | L-serine ammonia-lyase | -1.59 | 0.01 |
| RCC02558 | *hisD* | histidinol dehydrogenase | -1.63 | 0.02 |
| RCC03463 | *arcB2* | ornithine cyclodeaminase | -1.63 | 0 |
| RCC00797 | *hisG* | ATP phosphoribosyltransferase catalytic subunit | -1.67 | 0 |
| RCC02084 | *ltaE* | threonine aldolase | -1.7 | 0.002 |
| RCC01108 | *pyrroline-5-carboxylate reductase* | pyrroline-5-carboxylate reductase | -1.76 | 0.043 |
| RCC00866 | *argO* | arginine exporter protein ArgO | -1.75 | 0.032 |
| RCC01072 | *hutH* | histidine ammonia-lyase | -1.84 | 0.005 |
| RCC01128 | *trpF* | phosphoribosylanthranilate isomerase | -2.22 | 0 |
|  | | |  |  |
| **COG F: Nucleotide transport and metabolism** | | |  |  |
| RCC02617 | *ushA* | 5'-nucleotidase | 1.55 | 0.006 |
| RCC02778 | *class I glutamine amidotransferase* | class I glutamine amidotransferase | 1.53 | 0.007 |
| RCC02405 | *purU* | formyltetrahydrofolate deformylase | 1.49 | 0.016 |
| RCC01625 | *pyrH* | uridylate kinase | 1.46 | 0.012 |
| RCC01462 | *nrdJ2* | ribonucleoside-diphosphate reductase NrdJ | 1.42 | 0.004 |
| RCC00447 | *upp* | uracil phosphoribosyltransferase | 1.41 | 0.011 |
| RCC01720 | *ndk* | nucleoside diphosphate kinase | 1.4 | 0.018 |
| RCC02525 | *dht* | dihydropyrimidinase | 1.39 | 0.048 |
| RCC02168 | *purQ* | phosphoribosylformylglycinamidine synthase subunit | 1.38 | 0.037 |
| RCC01790 | *hpt* | hypoxanthine phosphoribosyltransferase | 1.35 | 0.015 |
| RCC02352 | *gpt* | xanthine phosphoribosyltransferase | 1.3 | 0.061 |
| RCC03041 | *thyX* | thymidylate synthase ThyX | 1.26 | 0.085 |
| RCC01914 | *dcd* | deoxycytidine triphosphate deaminase | -1.33 | 0.056 |
| RCC02956 | *nudF* | ADP-ribose pyrophosphatase | -1.46 | 0.007 |
| RCC01224 | *ureG* | urease accessory protein UreG | -1.6 | 0.046 |
| RCC01548 | *shikimate kinase domain-containing* | shikimate kinase domain-containing protein | -1.76 | 0.005 |
| RCC01251 | *tmk* | thymidylate kinase | -2.03 | 0 |
|  |  |  |  |  |
| **COG G: Carbohydrate transport and metabolism** | | |  |  |
| RCC02402 | *degP* | protease Do | 2.49 | 0 |
| RCC02877 | *ABC transporter* | K10555 AI-2 transport syste | 1.69 | 0 |
| RCC02657 | *ABC transporter* | K17202 erythritol transport | 1.61 | 0.008 |
| RCC03210 | *talC* | transaldolase | 1.48 | 0.006 |
| RCC01840 | *glgC* | glucose-1-phosphate adenylyltransferase | 1.48 | 0.026 |
| RCC02160 | *gap3* | glyceraldehyde-3-phosphate dehydrogenase | 1.43 | 0.008 |
| RCC02186 | *potF* | polyamine ABC transporter periplasmic | 1.43 | 0.027 |
| RCC01715 | *eno* | phosphopyruvate hydratase | 1.42 | 0.019 |
| RCC02373 | *ABC transporter* | K10543 D-xylose transport s | 1.42 | 0.035 |
| RCC02660 | *monosacharide ABC transporter permease* | K17203 erythritol transport system permease protein | 1.37 | 0.044 |
| RCC00579 | *cbbL* | ribulose bisphosphate carboxylase large subunit | 1.33 | 0.024 |
| RCC02482 | *HIT family protein* | Predicted Function | 1.29 | 0.05 |
| RCC01208 | *ppdK* | pyruvate, phosphate dikinase | -1.32 | 0.018 |
| RCC03235 | *monosacharide ABC transporter permease* | simple sugar transport system permease protein | -1.41 | 0.026 |
| RCC02544 | *ABC transporter substrate-binding protein* | multiple sugar transport system substrate-binding | -1.49 | 0.043 |
| RCC00787 | *monosacharide ABC transporter permease* | simple sugar transport system permease protein | -1.52 | 0.004 |
| RCC02407 | *pgl* | 6-phosphogluconolactonase | -1.53 | 0.019 |
| RCC03444 | *nagA* | N-acetylglucosamine-6-phosphate deacetylase | -1.53 | 0.019 |
| RCC01132 | *galM* | aldose 1-epimerase | -1.63 | 0.002 |
| RCC02027 | *mtlK* | mannitol 2-dehydrogenase | -1.64 | 0.003 |
| RCC03049 | *major facilitator superfamily protein* | Predicted Function | -1.67 | 0.012 |
| RCC00439 | *ppnk* | NAD(+) kinase | -1.7 | 0.002 |
| RCC00874 | *inositol monophosphatase* | inositol monophosphatase | -1.79 | 0.017 |
| RCC00856 | *nagZ* | beta-N-acetylhexosaminidase | -1.8 | 0.001 |
| RCC03443 | *BadF/BadG/BcrA/BcrD family ATPase* | BadF/BadG/BcrA/BcrD family ATPase | -2.12 | 0 |
|  |  |  |  |  |
| **COG H: Coenzyme transport and metabolism** | | |  |  |
| RCC00151 | *hemN1* | oxygen-independent coproporphyrinogen-III oxidase | 1.93 | 0 |
| RCC01034 | *CbiM family cobalamin biosynthesis protein* | cobalt/nickel transport system permease protein | 1.51 | 0.004 |
| RCC00836 | *cobS* | cobaltochelatase subunit CobS | 1.49 | 0.011 |
| RCC03531 | *ubiE* | ubiquinone/menaquinone biosynthesis methyltransferase | 1.46 | 0.003 |
| RCC00049 | *ahcY* | adenosylhomocysteinase | 1.46 | 0.002 |
| RCC00210 | *ubiG* | 3-demethylubiquinone-9 3-O-methyltransferase | 1.45 | 0.009 |
| RCC00384 | *gshA* | glutamate--cysteine ligase | 1.38 | 0.005 |
| RCC01120 | *metK* | methionine adenosyltransferase | 1.36 | 0.006 |
| RCC03435 | *molybdopterin binding domain* | Predicted Function | 1.35 | 0.039 |
| RCC03075 | *ribAB* | GTP cyclohydrolase II | 1.35 | 0.012 |
| RCC00195 | *hemH* | ferrochelatase | 1.34 | 0.016 |
| RCC01864 | *lipA* | lipoyl synthase | 1.32 | 0.031 |
| RCC00497 | *UbiA prenyltransferase* | UbiA prenyltransferase | -1.35 | 0.044 |
| RCC02360 | *moaC1* | molybdenum cofactor biosynthesis protein C | -1.36 | 0.014 |
| RCC02051 | *cobQ1* | cobyric acid synthase CobQ | -1.38 | 0.049 |
| RCC02038 | *cobF* | precorrin-6A synthase | -1.39 | 0.015 |
| RCC02053 | *cobC* | threonine-phosphate decarboxylase | -1.41 | 0.039 |
| RCC00712 | *moaA1* | molybdenum cofactor biosynthesis protein A | -1.43 | 0.004 |
| RCC02040 | *cobM* | precorrin-4 C11-methyltransferase | -1.51 | 0.049 |
| RCC02034 | *cbiO2* | cobalt ABC transporter ATP-binding protein CbiO | -1.51 | 0.015 |
| RCC02043 | *cobK* | precorrin-6x reductase | -1.51 | 0.019 |
| RCC03363 | *bioB* | biotin synthase | -1.57 | 0.032 |
| RCC00456 | *coaBC* | bifunctional phosphopantothenoylcysteine decarboxylase | -1.59 | 0.001 |
| RCC02044 | *cobJ* | precorrin-3B C17-methyltransferase | -1.6 | 0.017 |
| RCC00377 | *hemL* | glutamate-1-semialdehyde 2,1-aminomutase | -1.64 | 0.011 |
| RCC03354 | *cobQ2* | cobyric acid synthase CobQ | -1.64 | 0.016 |
| RCC02047 | *cobN* | cobaltochelatase subunit CobN | -1.65 | 0.002 |
| RCC02042 | *cobL* | precorrin-6Y C5,15-methyltransferase | -1.66 | 0.006 |
| RCC02430 | *nahG* | salicylate hydroxylase | -1.72 | 0.02 |
| RCC02041 | *cbiG* | cobalamin biosynthesis protein CbiG | -1.74 | 0.03 |
| RCC01734 | *ubiX* | 3-octaprenyl-4-hydroxybenzoate carboxy-lyase | -1.77 | 0.012 |
| RCC00878 | *5-formyltetrahydrofolate cyclo-ligase* | 5-formyltetrahydrofolate cyclo-ligase [EC | -1.79 | 0.032 |
| RCC03355 | *cobQ3* | cobyric acid synthase CobQ | -1.79 | 0.023 |
| RCC02036 | *cbiN* | cobalt transport protein CbiN | -1.85 | 0.018 |
| RCC02630 | *heme NO binding domain* | Predicted Function | -1.89 | 0 |
| RCC00072 | *nadB* | L-aspartate oxidase | -1.91 | 0.002 |
| RCC02059 | *cobV* | cobalamin 5'-phosphate synthase | -1.93 | 0.015 |
| RCC03364 | *bioF* | 8-amino-7-oxononanoate synthase | -1.96 | 0.009 |
|  |  |  |  |  |
| **COG I: Lipid transport and metabolism** | |  |  |  |
| RCC03306 | *acyl-CoA dehydrogenase* | acyl-CoA dehydrogenase, medium-chain specific | 1.85 | 0 |
| RCC01678 | *acpP1* | acyl carrier protein | 1.71 | 0 |
| RCC01634 | *fabZ* | (3R)-hydroxymyristoyl-ACP dehydratase | 1.7 | 0 |
| RCC02601 | *accB* | acetyl-CoA carboxylase biotin carboxyl carrier protein | 1.6 | 0 |
| RCC02468 | *thioesterase superfamily protein* | Predicted Function | 1.59 | 0 |
| RCC00538 | *methylmalonyl-CoA epimerase* | methylmalonyl-CoA/ethylmalonyl-CoA epimerase | 1.56 | 0.001 |
| RCC00746 | *phbC* | poly(3-hydroxyalkanoate) polymerase | 1.56 | 0.015 |
| RCC01791 | *cyclase/dehydrase* | cyclase/dehydrase | 1.52 | 0.037 |
| RCC02859 | *acsA3* | acetate--CoA ligase | 1.52 | 0.013 |
| RCC00236 | *accD* | acetyl-CoA carboxylase carboxyl transferase subunit beta | 1.52 | 0.01 |
| RCC02126 | *acsA1* | acetate--CoA ligase | 1.51 | 0.01 |
| RCC03178 | *atoB2* | acetyl-CoA acetyltransferase | 1.46 | 0.009 |
| RCC00678 | *crtA* | spheroidene monooxygenase | 1.46 | 0.002 |
| RCC00145 | *oxidoreductase* | Predicted Function | 1.44 | 0.016 |
| RCC00395 | *pgsA* | phosphatidyltransferase | 1.43 | 0.009 |
| RCC03241 | *mutB* | methylmalonyl-CoA mutase large subunit | 1.38 | 0.026 |
| RCC00679 | *crtI* | phytoene dehydrogenase | 1.3 | 0.026 |
| RCC01627 | *uppS* | di-trans,poly-cis-decaprenylcistransferase | -1.37 | 0.044 |
| RCC03449 | *atoB3* | acetyl-CoA acetyltransferase | -1.46 | 0.024 |
| RCC00696 | *dxs1* | 1-deoxy-D-xylulose-5-phosphate synthase | -1.47 | 0.012 |
| RCC03371 | *fabI* | enoyl-ACP reductase | -1.48 | 0.012 |
| RCC01870 | *fabF2* | 3-oxoacyl-ACP synthase II | -1.51 | 0.007 |
| RCC00520 | *fadB* | fatty acid oxidation complex subunit alpha | -1.51 | 0.009 |
| RCC00405 | *acyl-CoA dehydrogenase* | acyl-CoA dehydrogenase domain-containing protein | -1.55 | 0.032 |
| RCC00680 | *crtB* | phytoene synthase | -1.63 | 0.004 |
| RCC01794 | *pgpA* | phosphatidylglycerophosphatase A | -1.65 | 0.009 |
| RCC01063 | *pcl* | 4-coumarate--CoA ligase | -1.67 | 0.008 |
| RCC02796 | *pldB* | lysophospholipase L2 | -1.68 | 0.011 |
| RCC00461 | *est* | esterase | -1.68 | 0.01 |
| RCC01510 | *ivdH* | isovaleryl-CoA dehydrogenase | -2.08 | 0 |
| RCC01512 | *mccB* | methylcrotonoyl-CoA carboxylase subunit beta | -2.29 | 0 |
| RCC01516 | *menB* | naphthoate synthase | -2.31 | 0 |
| RCC01515 | *hmgL* | hydroxymethylglutaryl-CoA lyase | -2.52 | 0 |
| RCC01513 | *mccA* | methylcrotonoyl-CoA carboxylase subunit alpha | -2.54 | 0 |
|  |  |  |  |  |
| **COG J: Translation, ribosomal structure and biogenesis** | | |  |  |
| RCC02749 | *rpmG* | 50S ribosomal protein L33 | 1.89 | 0 |
| RCC02671 | *efp* | translation elongation factor P | 1.77 | 0.01 |
| RCC01205 | *glyQ* | glycyl-tRNA synthetase subunit alpha | 1.75 | 0.003 |
| RCC02550 | *infA* | translation initiation factor IF | 1.52 | 0.007 |
| RCC00281 | *rpsO* | 30S ribosomal protein S15 | 1.51 | 0.002 |
| RCC00309 | *rpsQ* | 30S ribosomal protein S17 | 1.48 | 0.018 |
| RCC00118 | *sigma 54 modulation protein* | sigma 54 modulation protein/ribosomal protein S30EA | 1.48 | 0.023 |
| RCC00298 | *rpsJ* | 30S ribosomal protein S10 | 1.48 | 0.009 |
| RCC03212 | *prmA* | ribosomal protein L11 methyltransferase | 1.48 | 0.003 |
| RCC00308 | *rpmC* | 50S ribosomal protein L29 | 1.47 | 0.018 |
| RCC00386 | *hypothetical protein* | 16S rRNA (uracil1498-N3)-methyltransferase | 1.47 | 0.029 |
| RCC02754 | *gatC* | glutamyl-tRNA(Gln) amidotransferase subunit C | 1.43 | 0.024 |
| RCC00313 | *rpsN* | 30S ribosomal protein S14 | 1.43 | 0.017 |
| RCC02464 | *proS* | prolyl-tRNA synthetase | 1.42 | 0.031 |
| RCC00303 | *rpsS* | 30S ribosomal protein S19 | 1.4 | 0.033 |
| RCC00314 | *rpsH* | 30S ribosomal protein S8 | 1.37 | 0.03 |
| RCC00284 | *pnp* | polyribonucleotide nucleotidyltransferase | 1.35 | 0.012 |
| RCC00324 | *rpsM* | 30S ribosomal protein S13 | 1.35 | 0.017 |
| RCC00316 | *rplR* | 50S ribosomal protein L18 | 1.35 | 0.044 |
| RCC00301 | *rplW* | 50S ribosomal protein L23 | 1.35 | 0.026 |
| RCC01125 | *rpsA* | 30S ribosomal protein S1 | 1.34 | 0.043 |
| RCC01711 | *tyrS* | tyrosyl-tRNA synthetase | 1.34 | 0.029 |
| RCC03153 | *pheS* | phenylalanyl-tRNA synthetase subunit alpha | 1.32 | 0.047 |
| RCC00841 | *gatB* | glutamyl-tRNA(Gln) amidotransferase subunit B | 1.32 | 0.024 |
| RCC00113 | *rnd1* | ribonuclease D | 1.31 | 0.015 |
| RCC01748 | *rsmB2* | ribosomal RNA small subunit methyltransferase B | -1.4 | 0.029 |
| RCC03094 | *rimJ* | ribosomal-protein-alanine N-acetyltransferase | -1.4 | 0.025 |
| RCC00189 | *cca* | CCA-adding enzyme | -1.53 | 0.008 |
| RCC00364 | *trmD* | tRNA (guanine-N1)-methyltransferase | -1.64 | 0.011 |
| RCC02784 | *TrmH family RNA methyltransferase* | 23S rRNA (guanosine2251-2'-O)-methyltransfera | -1.66 | 0.022 |
| RCC00191 | *tRNA (Uracil-5-)-methyltransferase* | 23S rRNA (uracil1939-C5)-methyltransferase | -1.69 | 0.006 |
| RCC00306 | *rplP* | 50S ribosomal protein L16 | -1.71 | 0.006 |
| RCC01796 | *dusB* | tRNA-dihydrouridine synthase B | -1.74 | 0.008 |
| RCC02506 | *rluB* | ribosomal large subunit pseudouridine synthase B | -1.76 | 0.004 |
| RCC03395 | *fmt* | methionyl-tRNA formyltransferase | -1.79 | 0.002 |
| RCC02470 | *selU* | tRNA 2-selenouridine synthase | -1.86 | 0.008 |
| RCC01961 | *gluQ* | glutamyl-Q tRNA(Asp) synthetase | -2.15 | 0 |
|  |  |  |  |  |
| **COG K: Transcription** | |  |  |  |
| RCC00458 | *rpoH1* | RNA polymerase sigma-32 factor | 1.74 | 0 |
| RCC00403 | *hypothetical protein* | putative transcriptional regulator | 1.68 | 0.023 |
| RCC00514 | *greA* | transcription elongation factor GreA | 1.65 | 0 |
| RCC01883 | *iscR* | FeS assembly cluster transcription factor IscR | 1.52 | 0 |
| RCC01010 | *DNA binding protein* | Ner family transcriptional regulator | 1.49 | 0.004 |
| RCC02811 | *rpoH2* | RNA polymerase sigma-32 factor | 1.46 | 0.05 |
| RCC02460 | *cspA2* | cold shock protein CspA | 1.45 | 0.004 |
| RCC01126 | *ihfB* | integration host factor subunit beta | 1.42 | 0.018 |
| RCC00326 | *rpoA* | DNA-directed RNA polymerase subunit alpha | 1.31 | 0.042 |
| RCC02361 | *lexA* | LexA repressor | 1.29 | 0.033 |
| RCC03311 | *rnc* | ribonuclease III | -1.38 | 0.039 |
| RCC00858 | *scpB* | segregation and condensation protein B | -1.52 | 0.002 |
| RCC03245 | *ROK family protein* | Predicted Function | -1.87 | 0 |
| RCC01737 | *oxyR* | hydrogen peroxide-inducible genes activator | -2.19 | 0.001 |
|  |  |  |  |  |
| **COG L: Replication, recombination and repair** | | |  |  |
| RCC02932 | *pinE* | DNA-invertase | 1.69 | 0.038 |
| RCC01001 | *bacteriophage DNA transposition B protein* | Predicted Function | 1.48 | 0.031 |
| RCC02567 | *resolvase* | resolvase | 1.47 | 0.037 |
| RCC00201 | *adenine-specific DNA-methyltransferase* | K13581 modification methylase | 1.44 | 0.038 |
| RCC01303 | *Cas2 family CRISPR-associated protein* | Predicted Function | 1.39 | 0.044 |
| RCC01249 | *TatD-related deoxyribonuclease* | TatD DNase family protein | 1.35 | 0.028 |
| RCC00146 | *parC* | DNA topoisomerase IV subunit A | -1.36 | 0.033 |
| RCC03211 | *priA* | primosomal protein N` | -1.38 | 0.041 |
| RCC01276 | *Cas1 family CRISPR-associated protein* | K15342 CRISP-associated protein Cas1 | -1.4 | 0.029 |
| RCC00255 | *polB* | DNA polymerase II | -1.41 | 0.025 |
| RCC02759 | *recR* | recombination protein RecR | -1.47 | 0.039 |
| RCC02007 | *phage integrase* | phage integrase | -1.48 | 0.046 |
| RCC02696 | *holC* | DNA polymerase III subunit chi | -1.48 | 0.032 |
| RCC03453 | *recQ* | ATP-dependent DNA helicase RecQ | -1.51 | 0.022 |
| RCC02429 | *xthA1* | exodeoxyribonuclease III | -1.52 | 0.008 |
| RCC00807 | *endonuclease/exonuclease/phosphatase* | endonuclease/exonuclease/phosphatase | -1.53 | 0.002 |
| RCC02318 | *yqgF* | holliday junction resolvase YqgF | -1.66 | 0.04 |
| RCC01499 | *DNA alkylation repair enzym* | DNA alkylation repair enzyme family protein | -1.78 | 0.001 |
| RCC01250 | *holB* | DNA polymerase III subunit delta' | -1.82 | 0.001 |
| RCC01665 | *recG* | ATP-dependent DNA helicase RecG | -1.93 | 0 |
| RCC03411 | *methyltransferase* | 16S rRNA (guanine966-N2)-methyltransferase | -1.98 | 0.002 |
| RCC00188 | *NUDIX superfamily hydrolase* | NUDIX superfamily hydrolase | -2.15 | 0 |
|  |  |  |  |  |
| **COG M: Cell wall/membrane/envelope biogenesis** | | |  |  |
| RCC01738 | *katG* | catalase/peroxidase | 2.19 | 0 |
| RCC00606 | *lipoprotein* | Predicted Function | 1.84 | 0.001 |
| RCC01179 | *transglycosylase, Slt family* | transglycosylase, Slt family | 1.83 | 0 |
| RCC02815 | *lipoprotein* | Predicted Function | 1.69 | 0 |
| RCC03326 | *lipoprotein* | lipoprotein | 1.67 | 0.001 |
| RCC00125 | *galU* | UTP--glucose-1-phosphate uridylyltransferase | 1.63 | 0.001 |
| RCC00908 | *lipoprotein* | Predicted Function | 1.62 | 0.002 |
| RCC03232 | *basic membrane lipoprotein family* | basic membrane protein A and related proteins | 1.57 | 0.003 |
| RCC01169 | *lipoprotein* | lipoprotein | 1.55 | 0.004 |
| RCC02490 | *OmpA family protein* | Predicted Function | 1.53 | 0.011 |
| RCC01578 | *lipoprotein* | Predicted Function | 1.53 | 0.016 |
| RCC00177 | *rfbC* | dTDP-4-dehydrorhamnose 3,5-epimerase | 1.53 | 0.002 |
| RCC01873 | *peptidoglycan-binding domain 1 protein* | peptidoglycan-binding domain 1 protein | 1.37 | 0.012 |
| RCC01209 | *cell wall hydrolase, SleB* | cell wall hydrolase, SleB | 1.35 | 0.046 |
| RCC03390 | *VacJ family lipoprotein* | lipoprotein | 1.32 | 0.018 |
| RCC00176 | *rfbB* | dTDP-glucose 4,6-dehydratase | 1.29 | 0.044 |
| RCC01496 | *lipoprotein* | lipoprotein | -1.36 | 0.04 |
| RCC01247 | *membrane transport family protein* | membrane transport family protein | -1.38 | 0.034 |
| RCC02961 | *family 25 glycosyl transferase* | glycosyl transferase, family 25 | -1.41 | 0.003 |
| RCC00114 | *kdsD* | arabinose 5-phosphate isomerase | -1.42 | 0.035 |
| RCC00269 | *lipoprotein* | lipoprotein | -1.44 | 0.039 |
| RCC02510 | *lipoprotein* | lipoprotein | -1.47 | 0.018 |
| RCC03465 | *membrane transport family protein* | membrane transport family protein | -1.48 | 0.025 |
| RCC03450 | *pbpC* | penicillin-binding protein 1C | -1.48 | 0.022 |
| RCC02627 | *dacB* | penicillin-binding protein 4 | -1.5 | 0.018 |
| RCC03195 | *OmpA/MotB domain-containing protein* | peptidoglycan-associated lipoprotein | -1.52 | 0.027 |
| RCC01637 | *lpxB* | lipid-A-disaccharide synthase | -1.53 | 0.011 |
| RCC01119 | *lnt* | apolipoprotein N-acyltransferase | -1.53 | 0.005 |
| RCC01115 | *lipoprotein* | lipoprotein | -1.53 | 0.026 |
| RCC00229 | *lpxK* | tetraacyldisaccharide 4'-kinase | -1.54 | 0.022 |
| RCC00174 | *rfbD* | dTDP-4-dehydrorhamnose reductase | -1.54 | 0.007 |
| RCC00642 | *peptidoglycan binding domain* | peptidoglycan binding domain-containing protein | -1.55 | 0.012 |
| RCC01114 | *OmpA/MotB domain-containing protein* | OmpA/MotB domain-containing protein | -1.57 | 0.006 |
| RCC02427 | *M23 family peptidase* | M23 family peptidase | -1.58 | 0.015 |
| RCC02704 | *NLP/P60 family protein* | Predicted Function | -1.58 | 0.027 |
| RCC00722 | *lipoprotein* | Predicted Function | -1.63 | 0.015 |
| RCC00734 | *lipoprotein* | Predicted Function | -1.74 | 0.015 |
| RCC01381 | *lipoprotein* | lipoprotein | -1.74 | 0.001 |
| RCC00140 | *lipoprotein* | K14160 protein ImuA | -1.76 | 0.002 |
| RCC00172 | *glycosyl transferase* | glycosyl transferase | -1.83 | 0.001 |
| RCC00256 | *lipoprotein* | LPS-assembly lipoprotein | -1.85 | 0 |
| RCC02693 | *permease YjgP/YjgQ family protein* | K11720 lipopolysaccharide export system permease protein | -1.86 | 0.004 |
| RCC00649 | *dgkA* | diacylglycerol kinase | -1.88 | 0.002 |
| RCC02751 | *N-acetylmuramoyl-L-alanine amidase* | N-acetylmuramoyl-L-alanine amidase, family 2 | -2.3 | 0 |
| RCC02596 | *lipoprotein* | Predicted Function | -2.45 | 0 |
|  |  |  |  |  |
| **COG N: Cell motility** | |  |  |  |
| RCC01353 | *cheA1* | chemotaxis protein CheA | -1.35 | 0.043 |
| RCC02075 | *PAS/PAC sensor domain- protein* | aerotaxis receptor | -1.37 | 0.011 |
| RCC03176 | *PAS/PAC sensor domain- protein* | aerotaxis receptor | -1.44 | 0.002 |
| RCC00010 | *flgI* | flagellar P-ring protein FlgI | -1.5 | 0.015 |
| RCC02887 | *methyl-accepting chemotaxis transducer* | methyl-accepting chemotaxis sensory transducer | -1.51 | 0.044 |
| RCC02139 | *methyl-accepting chemotaxis protein* | Predicted Function | -1.58 | 0.012 |
| RCC03521 | *fliI* | flagellar protein export ATPase FliI | -1.61 | 0.029 |
| RCC01621 | *mcpC* | methyl-accepting chemotaxis protein McpC | -1.63 | 0.003 |
| RCC02611 | *mcpA3* | methyl-accepting chemotaxis protein McpA | -1.64 | 0 |
| RCC00644 | *mcpX* | methyl-accepting chemotaxis protein McpX | -1.71 | 0.005 |
| RCC01051 | *gvpN* | gas vesicle protein GvpN | -1.77 | 0 |
| RCC03514 | *flgA* | flagella basal body P-ring formation protein FlgA | -1.81 | 0.009 |
| RCC01667 | *methyl-accepting chemotaxis* | methyl-accepting chemotaxis sensory transducer | -1.83 | 0 |
| RCC01054 | *gvpJ* | gas vesicle protein GvpJ | -1.93 | 0.001 |
| RCC01056 | *gas vesicle protein GvpL/GvpF* | gas vesicle synthesis protein GvpL/GvpF | -2.31 | 0 |
|  |  |  |  |  |
| **COG O: Post-translational modification, protein turnover, and chaperones** | | |  |  |
| RCC02477 | *groS* | chaperonin GroS | 1.87 | 0 |
| RCC00228 | *DSBA family oxidoreductase* | DSBA family oxidoreductase | 1.74 | 0.006 |
| RCC00838 | *DnaJ domain-containing protein* | Predicted Function | 1.65 | 0 |
| RCC02818 | *ibpA* | small heat shock protein IbpA | 1.65 | 0.012 |
| RCC03325 | *hemimethylated DNA-binding protein* | K11940 heat shock protein HspQ | 1.64 | 0.003 |
| RCC00224 | *dnaK* | chaperone DnaK | 1.59 | 0 |
| RCC02478 | *groL* | chaperonin GroL | 1.55 | 0.003 |
| RCC00547 | *glutaredoxin family protein* | monothiol glutaredoxin | 1.55 | 0.007 |
| RCC03257 | *msrA1* | peptide-methionine-(S)-S-oxide reductase | 1.54 | 0.009 |
| RCC02008 | *tig* | trigger factor | 1.54 | 0.016 |
| RCC00035 | *hslV* | ATP-dependent protease HslV | 1.51 | 0.001 |
| RCC03406 | *clpB* | chaperone ClpB | 1.49 | 0.013 |
| RCC02609 | *clpP* | ATP-dependent Clp protease proteolytic subunit | 1.47 | 0.014 |
| RCC03175 | *dsbE* | thiol:disulfide interchange protein | 1.45 | 0.024 |
| RCC02400 | *hflC* | HflC protein | 1.41 | 0.014 |
| RCC00036 | *trxA1* | thioredoxin | 1.36 | 0.003 |
| RCC00034 | *hslU* | ATP-dependent hsl protease ATP-binding subunit | 1.36 | 0.015 |
| RCC01743 | *DSBA family oxidoreductase* | DSBA family oxidoreductase | 1.36 | 0.012 |
| RCC01710 | *ppiB* | peptidyl-prolyl cis-trans isomerase B | 1.29 | 0.023 |
| RCC01786 | *ccmB* | heme exporter protein B | -1.35 | 0.025 |
| RCC01785 | *ccmA* | heme exporter protein A | -1.54 | 0.043 |
| RCC00334 | *ATP12 chaperone protein family* | Predicted Function | -1.61 | 0.019 |
| RCC01217 | *ureD* | urease accessory protein UreD | -1.66 | 0.032 |
| RCC02893 | *cspA3* | cold shock protein CspA | -1.66 | 0.002 |
| RCC02426 | *pcm1* | protein-L-isoaspartate O-methyltransferase | -1.69 | 0.007 |
| RCC02691 | *surA* | chaperone SurA | -1.7 | 0.007 |
| RCC00607 | *cspA1* | cold shock protein CspA | -1.77 | 0.002 |
| RCC00806 | *DnaJ domain-containing protein* | heat shock protein DnaJ domain-containing protein | -1.86 | 0 |
| RCC02868 | *S1/S6 family peptidase* | S1/S6 family peptidase | -1.92 | 0 |
| RCC01514 | *glutathione S-transferase* | glutathione S-transferase | -2.01 | 0.001 |
| RCC00762 | *hoxW* | hydrogenase maturation factor | -2.23 | 0.001 |
|  |  |  |  |  |
| **COG P: Inorganic ion transport and metabolism** | | |  |  |
| RCC02219 | *modA2* | molybdate ABC transporter periplasmic molybdate | 4.9 | 0 |
| RCC02220 | *modC2* | molybdate ABC transporter ATP-binding protein | 4.06 | 0 |
| RCC02218 | *modB2* | molybdate ABC transporter permease | 2.28 | 0.002 |
| RCC00563 | *modB1* | molybdenum ABC transporter permease | 2.18 | 0 |
| RCC02789 | *kefC1* | glutathione-regulated potassium-efflux system protein | 1.82 | 0 |
| RCC02861 | *actP3* | cation/acetate symporter ActP | 1.74 | 0.002 |
| RCC02124 | *actP2* | cation/acetate symporter ActP | 1.71 | 0.003 |
| RCC00585 | *anfH* | nitrogenase iron protein | 1.72 | 0.011 |
| RCC03264 | *NifZ family protein* | nitrogen fixation protein NifZ | 1.69 | 0.04 |
| RCC02311 | *sodB* | superoxide dismutase | 1.65 | 0.006 |
| RCC02476 | *ppaC* | inorganic pyrophosphatase | 1.62 | 0.006 |
| RCC00572 | *nifH1* | nitrogenase iron protein | 1.53 | 0.047 |
| RCC00376 | *divalent ion symporter* | divalent ion symporter | 1.51 | 0.001 |
| RCC01895 | *potH1* | polyamine ABC transporter permease PotH | 1.51 | 0.018 |
| RCC00913 | *bfr* | bacterioferritin | 1.47 | 0.022 |
| RCC02132 | *phaAB* | monovalent cation/proton antiporter subunit A/B | 1.46 | 0.038 |
| RCC00564 | *modC1* | molybdenum ABC transporter ATP-binding protein | 1.42 | 0.009 |
| RCC02707 | *cynT* | carbonate dehydratase | 1.42 | 0.009 |
| RCC03386 | *amtB* | ammonium transporter | 1.4 | 0.005 |
| RCC00375 | *mdoG1* | glucans biosynthesis protein G | 1.38 | 0.032 |
| RCC02521 | *pyrimidine ABC transporter* | NitT/TauT family transport | 1.36 | 0.047 |
| RCC01599 | *iscA* | iron-sulfur cluster assembly accessory protein | 1.34 | 0.046 |
| RCC01894 | *potI2* | polyamine ABC transporter permease PotI | 1.33 | 0.045 |
| RCC01811 | *major facilitator superfamily protein* | DHA1 family, bicyclomycin/chloramphen | 1.29 | 0.046 |
| RCC00789 | *ABC transporter* | monosacharide ABC transporter ATP-binding protein | -1.3 | 0.026 |
| RCC01180 | *copA2* | copper-transporting P-type ATPase | -1.4 | 0.039 |
| RCC02130 | *phaD* | monovalent cation/proton antiporter subunit D | -1.42 | 0.025 |
| RCC01163 | *ccoI* | cbb3-type cytochrome c oxidase biogenesis protein CcoI | -1.5 | 0.003 |
| RCC00576 | *cbbO* | rubisco activation protein CbbO | -1.61 | 0.002 |
| RCC03015 | *kefC2* | glutathione-regulated potassium-efflux system protein | -1.65 | 0.002 |
| RCC03251 | *cbiO3* | cobalt ABC transporter ATP-binding protein CbiO | -1.66 | 0.029 |
| RCC02028 | *FeoA family protein* | ferrous iron transport protein A | -1.72 | 0.025 |
| RCC00904 | *major facilitator superfamily protein* | DHA1 family, bicyclomycin/chloramphen | -1.76 | 0.009 |
| RCC02116 | *iron siderophore/cobalamin ABC transporter* | iron complex tra | -1.85 | 0.002 |
| RCC01136 | *znuB* | zinc ABC transporter permease ZnuB | -1.99 | 0.007 |
| RCC02035 | *cbiQ2* | cobalt ABC transporter permease CbiQ | -2.23 | 0.001 |
|  |  |  |  |  |
| **COG Q: Secondary metabolites biosynthesis, transport, and catabolism** | | |  |  |
| RCC01368 | *antibiotic biosynthesis monooxygenase* | antibiotic biosynthesis monooxygenase | 1.98 | 0 |
| RCC03256 | *cytochrome P450 family protein* | cytochrome P450 family protein | 1.87 | 0 |
| RCC02882 | *antibiotic biosynthesis monooxygenase* | K11530 autoinducer 2-degrading protein | 1.75 | 0.002 |
| RCC01376 | *dctP1* | TRAP C4-dicarboxylate transport system permease subunit | 1.61 | 0.007 |
| RCC01544 | *dctQ2* | TRAP C4-dicarboxylate transport system permease subunit | 1.59 | 0.001 |
| RCC02824 | *DSBA family oxidoreductase* | DSBA family oxidoreductase | 1.57 | 0.009 |
| RCC02417 | *dctP2* | TRAP C4-dicarboxylate transport system permease subunit | 1.51 | 0.007 |
| RCC00158 | *M10 family peptidase* | M10 family peptidase | 1.35 | 0.047 |
| RCC02016 | *fumarylacetoacetate hydrolase* | fumarylacetoacetate hydrolase | 1.34 | 0.041 |
| RCC01145 | *fabH1* | 3-oxoacyl-ACP synthase III | 1.3 | 0.016 |
| RCC01545 | *dctM2* | TRAP C4-dicarboxylate transport system permease subunit | 1.27 | 0.05 |
| RCC02509 | *TelA family toxic anion resistance protein* | TelA family toxic anion resistance protein | -1.34 | 0.02 |
| RCC01573 | *ABC transporter permease* | putative ABC transport system permease protein | -1.36 | 0.025 |
| RCC00861 | *thioesterase* | thioesterase | -1.62 | 0.038 |
| RCC03378 | *CDA peptide synthetase III* | CDA peptide synthetase III | -1.67 | 0 |
| RCC00148 | *CDA peptide synthetase III* | CDA peptide synthetase III | -1.75 | 0 |
|  |  |  |  |  |
| **COG R: General function prediction only** | | |  |  |
| RCC01589 | *ErfK/YbiS/YcfS/YnhG family protein* | ErfK/YbiS/YcfS/YnhG family protein | 3.08 | 0 |
| RCC00938 | *phage terminase large subunit* | phage terminase large subunit | 2.74 | 0 |
| RCC00194 | *ErfK/YbiS/YcfS/YnhG family protein* | ErfK/YbiS/YcfS/YnhG family protein | 1.83 | 0.002 |
| RCC00880 | *CHAP domain-containing protein* | CHAP domain-containing protein | 1.83 | 0.002 |
| RCC00975 | *cyclic nucleotide domain* | cyclic nucleotide-binding domain-containing protein | 1.81 | 0.022 |
| RCC00998 | *gam* | host-nuclease inhibitor protein Gam | 1.77 | 0.001 |
| RCC02755 | *metallo-beta-lactamase* | metallo-beta-lactamase | 1.72 | 0.001 |
| RCC03248 | *metallophosphoesterase* | metallophosphoesterase | 1.63 | 0.01 |
| RCC01184 | *CsbD family protein* | CsbD family protein | 1.62 | 0.006 |
| RCC03426 | *AMP-dependent synthetase and ligase* | long-chain acyl-CoA synthetase | 1.54 | 0.001 |
| RCC02612 | *transthyretin family protein* | 5-hydroxyisourate hydrolase | 1.51 | 0.007 |
| RCC01884 | *alpha/beta fold family hydrolase* |  | 1.5 | 0.005 |
| RCC02325 | *HesB/YadR/YfhF family protein* | HesB/YadR/YfhF family protein | 1.49 | 0.008 |
| RCC00936 | *lambda family phage portal protein* | lambda family phage portal protein | 1.48 | 0.024 |
| RCC03416 | *endoribonuclease L-PSP family protein* | endoribonuclease L-PSP family protein | 1.43 | 0.007 |
| RCC03490 | *endoribonuclease, L-PSP family* | endoribonuclease, L-PSP family | 1.41 | 0.027 |
| RCC02390 | *alkane 1-monooxygenase* | alkane 1-monooxygenase | 1.37 | 0.042 |
| RCC00128 | *ABC transporter permease* | ABC transporter permease | 1.36 | 0.045 |
| RCC03456 | *major facilitator superfamily protein* | MFS transporter, UMF1 family | 1.33 | 0.035 |
| RCC00192 | *ErfK/YbiS/YcfS/YnhG family protein* | ErfK/YbiS/YcfS/YnhG family protein | 1.26 | 0.047 |
| RCC00529 | *FAD dependent oxidoreductase* | FAD dependent oxidoreductase | -1.31 | 0.041 |
| RCC01825 | *HAD superfamily hydrolase* | HAD superfamily hydrolase | -1.36 | 0.016 |
| RCC03410 | *HAD superfamily hydrolase* | HAD superfamily hydrolase | -1.39 | 0.038 |
| RCC00816 | *extradiol ring-cleavage dioxygenase subunit B* | extradiol ring-cleavage dioxygenase subunit B | -1.4 | 0.016 |
| RCC00333 | *gph1* | phosphoglycolate phosphatase | -1.47 | 0.01 |
| RCC00464 | *tetrapyrrole methylase* | 16S rRNA (cytidine1402-2'-O)-methyltransferase | -1.48 | 0.022 |
| RCC02196 | *FkbM family methyltransferase* | FkbM family methyltransferase | -1.48 | 0.041 |
| RCC02435 | *family 2 glycosyl transferase* | family 2 glycosyl transferase | -1.54 | 0.017 |
| RCC01235 | *pyrimidine 5'-nucleotidase* | putative hydrolase of the HAD superfamily | -1.57 | 0.039 |
| RCC02991 | *hadH* | 3-hydroxy-2-methylbutyryl-CoA dehydrogenase | -1.62 | 0.024 |
| RCC01687 | *HK97 family phage major capsid protein* | HK97 family phage major capsid protein | -1.63 | 0.043 |
| RCC02194 | *phospholipase/carboxylesterase* | phospholipase/carboxylesterase | -1.64 | 0.001 |
| RCC02068 | *ice nucleation protein repeat family protein* | ice nucleation protein repeat family protein | -1.66 | 0.039 |
| RCC03246 | *family 2 glycosyl transferase* | family 2 glycosyl transferase | -1.67 | 0.021 |
| RCC00629 | *GNAT family acetyltransferase* | phosphinothricin acetyltransferase | -1.68 | 0.02 |
| RCC01559 | *type 11 family methyltransferase* | type 11 family methyltransferase | -1.73 | 0.014 |
| RCC00519 | *cupin domain-containing protein* | cupin domain-containing protein | -1.74 | 0.016 |
| RCC00406 | *metallo-beta-lactamase* | metallo-beta-lactamase | -1.77 | 0.016 |
| RCC02362 | *competence protein* | competence protein ComEC | -1.79 | 0.011 |
| RCC02297 | *GNAT family acetyltransferase* | phosphinothricin acetyltransferase | -1.94 | 0.009 |
| RCC01406 | *phage lysozyme* | lysozyme | -2.19 | 0.001 |
| RCC02983 | *extensin family protein* | extensin family protein | -2.33 | 0 |
| RCC02639 | *NUDIX superfamily hydrolase* | NUDIX superfamily hydrolase | -2.35 | 0.001 |
|  |  |  |  |  |
| **COG S: Function unknown** | |  |  |  |
| RCC03108 *hypothetical protein* | | Unknown Function | 3.25 | 0 |
| RCC02462 | *hypothetical protein* | Unknown Function | 2.78 | 0 |
| RCC00890 | *hypothetical protein* | Unknown Function | 2.6 | 0 |
| RCC00423 | *hypothetical protein* | Unknown Function | 2.47 | 0 |
| RCC01268 | *hypothetical protein* | Unknown Function | 2.42 | 0 |
| RCC02154 | *TM2 domain-containing protein* | TM2 domain-containing protein | 2.4 | 0 |
| RCC03304 | *hypothetical protein* | Unknown Function | 2.39 | 0 |
| RCC00353 | *hypothetical protein* | Unknown Function | 2.3 | 0 |
| RCC00871 | *hypothetical protein* | hypothetical protein | 2.26 | 0 |
| RCC03009 | *hypothetical protein* | Unknown Function | 2.2 | 0 |
| RCC02709 | *hypothetical protein* | Unknown Function | 2.14 | 0 |
| RCC02860 | *hypothetical protein* | Unknown Function | 1.98 | 0.001 |
| RCC00737 | *hypothetical protein* | Unknown Function | 1.98 | 0.001 |
| RCC02923 | *hypothetical protein* | Unknown Function | 1.96 | 0.001 |
| RCC02125 | *hypothetical protein* | Unknown Function | 1.94 | 0.001 |
| RCC01112 | *hypothetical protein* | Unknown Function | 1.92 | 0.005 |
| RCC00050 | *hypothetical protein* | Unknown Function | 1.92 | 0.001 |
| RCC02924 | *hypothetical protein* | Unknown Function | 1.91 | 0.017 |
| RCC01874 | *hypothetical protein* | Unknown Function | 1.81 | 0 |
| RCC00981 | *phage head morphogenesis protein* | phage head morphogenesis protein | 1.81 | 0.005 |
| RCC03008 | *hypothetical protein* | Unknown Function | 1.8 | 0.001 |
| RCC03343 | *hypothetical protein* | Unknown Function | 1.78 | 0.007 |
| RCC02553 | *hypothetical protein* | Unknown Function | 1.77 | 0.001 |
| RCC02808 | *hypothetical protein* | Unknown Function | 1.77 | 0 |
| RCC01739 | *hypothetical protein* | Unknown Function | 1.72 | 0 |
| RCC00066 | *hypothetical protein* | putative membrane protein | 1.72 | 0.004 |
| RCC00231 | *hypothetical protein* | Unknown Function | 1.7 | 0 |
| RCC02568 | *hypothetical protein* | Unknown Function | 1.7 | 0.003 |
| RCC02710 | *hypothetical protein* | Unknown Function | 1.7 | 0 |
| RCC00293 | *hypothetical protein* | Unknown Function | 1.67 | 0.001 |
| RCC00991 | *hypothetical protein* | Unknown Function | 1.67 | 0.024 |
| RCC03124 | *hypothetical protein* | Unknown Function | 1.67 | 0.01 |
| RCC01012 | *hypothetical protein* | Unknown Function | 1.63 | 0.001 |
| RCC00989 | *lysozyme* | lysozyme | 1.62 | 0.046 |
| RCC01423 | *hypothetical protein* | Unknown Function | 1.62 | 0.003 |
| RCC02921 | *hypothetical protein* | Unknown Function | 1.6 | 0.032 |
| RCC01622 | *hypothetical protein* | Unknown Function | 1.59 | 0.004 |
| RCC00977 | *hypothetical protein* | Unknown Function | 1.59 | 0.043 |
| RCC02656 | *hypothetical protein* | Unknown Function | 1.58 | 0.009 |
| RCC02587 | *hypothetical protein* | Unknown Function | 1.58 | 0.001 |
| RCC00150 | *hypothetical protein* | hypothetical protein | 1.58 | 0.041 |
| RCC01038 | *hypothetical protein* | Unknown Function | 1.58 | 0.036 |
| RCC00543 | *hypothetical protein* | Unknown Function | 1.57 | 0.003 |
| RCC03143 | *hypothetical protein* | Unknown Function | 1.57 | 0.019 |
| RCC01013 | *hypothetical protein* | Unknown Function | 1.57 | 0.003 |
| RCC00884 | *hypothetical protein* | Unknown Function | 1.57 | 0.022 |
| RCC02346 | *hypothetical protein* | Unknown Function | 1.56 | 0.003 |
| RCC01814 | *hypothetical protein* | Unknown Function | 1.56 | 0.003 |
| RCC02939 | *hypothetical protein* | hypothetical protein | 1.56 | 0.016 |
| RCC00935 | *hypothetical protein* | Unknown Function | 1.55 | 0.005 |
| RCC03181 | *hypothetical protein* | Unknown Function | 1.55 | 0.015 |
| RCC03242 | *hypothetical protein* | Unknown Function | 1.53 | 0.024 |
| RCC00995 | *hypothetical protein* | Unknown Function | 1.53 | 0.014 |
| RCC01584 | *hypothetical protein* | Unknown Function | 1.53 | 0.014 |
| RCC02817 | *hypothetical protein* | Unknown Function | 1.52 | 0.02 |
| RCC01254 | *phage integrase* | phage integrase | 1.52 | 0.025 |
| RCC03399 | *hypothetical protein* | Unknown Function | 1.51 | 0.013 |
| RCC02391 | *hypothetical protein* | Unknown Function | 1.5 | 0.039 |
| RCC01463 | *hypothetical protein* | Unknown Function | 1.5 | 0.01 |
| RCC02828 | *hypothetical protein* | Unknown Function | 1.5 | 0.025 |
| RCC01666 | *hypothetical protein* | Unknown Function | 1.5 | 0.013 |
| RCC03320 | *hypothetical protein* | Unknown Function | 1.5 | 0.001 |
| RCC00949 | *hypothetical protein* | Unknown Function | 1.49 | 0.023 |
| RCC02676 | *hypothetical protein* | Unknown Function | 1.49 | 0.008 |
| RCC02288 | *hypothetical protein* | Unknown Function | 1.49 | 0.028 |
| RCC00914 | *hypothetical protein* | Unknown Function | 1.48 | 0.047 |
| RCC03417 | *hypothetical protein* | Unknown Function | 1.48 | 0.008 |
| RCC01869 | *invasion associated locus B family protein* | Unknown Function | 1.48 | 0.023 |
| RCC03207 | *hypothetical protein* | Unknown Function | 1.45 | 0.013 |
| RCC02012 | *hypothetical protein* | Unknown Function | 1.44 | 0.012 |
| RCC02933 | *hypothetical protein* | Unknown Function | 1.44 | 0.044 |
| RCC00575 | *hypothetical protein* | Unknown Function | 1.43 | 0.02 |
| RCC00052 | *hypothetical protein* | Unknown Function | 1.42 | 0.039 |
| RCC01921 | *hypothetical protein* | Unknown Function | 1.42 | 0.016 |
| RCC02301 | *hypothetical protein* | Unknown Function | 1.42 | 0.005 |
| RCC01725 | *hypothetical protein* | Unknown Function | 1.41 | 0.001 |
| RCC03379 | *hypothetical protein* | Unknown Function | 1.41 | 0.014 |
| RCC02078 | *hypothetical protein* | Unknown Function | 1.4 | 0.043 |
| RCC03305 | *hypothetical protein* | Unknown Function | 1.4 | 0.026 |
| RCC00133 | *hypothetical protein* | Unknown Function | 1.39 | 0.031 |
| RCC02344 | *hypothetical protein* | Unknown Function | 1.38 | 0.05 |
| RCC01677 | *hypothetical protein* | Unknown Function | 1.38 | 0.013 |
| RCC01014 | *hypothetical protein* | Unknown Function | 1.37 | 0.026 |
| RCC00227 | *hypothetical protein* | Unknown Function | 1.37 | 0.019 |
| RCC01312 | *hypothetical protein* | Unknown Function | 1.37 | 0.035 |
| RCC00429 | *hypothetical protein* | Unknown Function | 1.37 | 0.05 |
| RCC03069 | *hypothetical protein* | Unknown Function | 1.37 | 0.044 |
| RCC01522 | *hypothetical protein* | Unknown Function | 1.37 | 0.032 |
| RCC03454 | *hypothetical protein* | YggT family protein | 1.36 | 0.045 |
| RCC00542 | *hypothetical protein* | Unknown Function | 1.35 | 0.039 |
| RCC00905 | *hypothetical protein* | Unknown Function | 1.33 | 0.035 |
| RCC01263 | *hypothetical protein* | Unknown Function | 1.32 | 0.043 |
| RCC02155 | *hypothetical protein* | K14205 phosphatidylglycerol lysyltransferase | -1.27 | 0.043 |
| RCC00120 | *hypothetical protein* | Unknown Function | -1.31 | 0.027 |
| RCC00059 | *hypothetical protein* | Unknown Function | -1.34 | 0.021 |
| RCC01741 | *hypothetical protein* | hypothetical protein | -1.36 | 0.015 |
| RCC02072 | *nonfunctional major facilitator* | nonfunctional major facilitator superfamily protein | -1.37 | 0.018 |
| RCC02560 | *hypothetical protein* | Unknown Function | -1.38 | 0.041 |
| RCC03394 | *hypothetical protein* | Unknown Function | -1.38 | 0.029 |
| RCC03205 | *hypothetical protein* | Unknown Function | -1.39 | 0.019 |
| RCC00389 | *hypothetical protein* | Unknown Function | -1.39 | 0.029 |
| RCC02603 | *hypothetical protein* | Unknown Function | -1.4 | 0.028 |
| RCC00500 | *hypothetical protein* | Unknown Function | -1.4 | 0.035 |
| RCC00596 | *hypothetical protein* | Unknown Function | -1.41 | 0.01 |
| RCC01610 | *hypothetical protein* | Unknown Function | -1.43 | 0.005 |
| RCC03148 | *hypothetical protein* | Unknown Function | -1.44 | 0.03 |
| RCC00881 | *metallophosphoesterase* | hypothetical protein | -1.44 | 0.015 |
| RCC02512 | *hypothetical protein* | Unknown Function | -1.44 | 0.003 |
| RCC03487 | *hypothetical protein* | Unknown Function | -1.46 | 0.021 |
| RCC03341 | *hypothetical protein* | Unknown Function | -1.47 | 0.031 |
| RCC02485 | *type 12 family methyltransferase* | type 12 family methyltransferase | -1.48 | 0.025 |
| RCC03118 | *hypothetical protein* | Unknown Function | -1.48 | 0.031 |
| RCC00794 | *SlyX family protein* | SlyX protein | -1.49 | 0.036 |
| RCC01140 | *hypothetical protein* | Unknown Function | -1.49 | 0.009 |
| RCC02703 | *hypothetical protein* | Unknown Function | -1.5 | 0.015 |
| RCC00175 | *hypothetical protein* | Unknown Function | -1.5 | 0.003 |
| RCC02153 | *SCP-like extracellular protein family* | SCP-like extracellular protein family | -1.5 | 0.043 |
| RCC03167 | *hypothetical protein* | cell division transport system permease protein | -1.5 | 0.05 |
| RCC00541 | *hypothetical protein* | hypothetical protein | -1.51 | 0.04 |
| RCC01350 | *hypothetical protein* | Unknown Function | -1.51 | 0.012 |
| RCC03053 | *hypothetical protein* | Unknown Function | -1.51 | 0.032 |
| RCC03528 | *hypothetical protein* | Unknown Function | -1.51 | 0.041 |
| RCC01585 | *hypothetical protein* | Unknown Function | -1.51 | 0.009 |
| RCC00909 | *hypothetical protein* | Unknown Function | -1.52 | 0.025 |
| RCC00084 | *hypothetical protein* | Unknown Function | -1.54 | 0.009 |
| RCC01359 | *hypothetical protein* | Unknown Function | -1.54 | 0.014 |
| RCC01455 | *hypothetical protein* | aspartyl protease family protein | -1.56 | 0.006 |
| RCC03506 | *YCII-related domain-containing protein* | hypothetical protein | -1.56 | 0.021 |
| RCC02069 | *hypothetical protein* | Unknown Function | -1.56 | 0.02 |
| RCC00658 | *hypothetical protein* | Unknown Function | -1.57 | 0.004 |
| RCC01714 | *hypothetical protein* | hypothetical protein | -1.58 | 0.034 |
| RCC00307 | *hypothetical protein* | Unknown Function | -1.58 | 0.043 |
| RCC01877 | *hypothetical protein* | Unknown Function | -1.58 | 0.003 |
| RCC01139 | *hypothetical protein* | Unknown Function | -1.59 | 0 |
| RCC00238 | *hypothetical protein* | hypothetical protein | -1.6 | 0.032 |
| RCC03052 | *hypothetical protein* | Unknown Function | -1.6 | 0.004 |
| RCC02588 | *hypothetical protein* | Unknown Function | -1.6 | 0.02 |
| RCC00448 | *sporulation domain-containing protein* | sporulation domain-containing protein | -1.62 | 0.012 |
| RCC00612 | *hypothetical protein* | hypothetical protein | -1.62 | 0.028 |
| RCC00499 | *hypothetical protein* | Unknown Function | -1.62 | 0.04 |
| RCC03332 | *hypothetical protein* | hypothetical protein | -1.63 | 0.025 |
| RCC03160 | *hypothetical protein* | Unknown Function | -1.64 | 0.026 |
| RCC03440 | *hypothetical protein* | Unknown Function | -1.64 | 0.001 |
| RCC01080 | *hypothetical protein* | Unknown Function | -1.64 | 0.026 |
| RCC02263 | *hypothetical protein* | Unknown Function | -1.64 | 0.03 |
| RCC00546 | *hypothetical protein* | Unknown Function | -1.64 | 0.028 |
| RCC02293 | *hypothetical protein* | Unknown Function | -1.64 | 0.025 |
| RCC02147 | *hypothetical protein* | Unknown Function | -1.64 | 0.016 |
| RCC02651 | *hypothetical protein* | Unknown Function | -1.65 | 0.042 |
| RCC02772 | *hypothetical protein* | Unknown Function | -1.65 | 0.002 |
| RCC00244 | *hypothetical protein* | Unknown Function | -1.66 | 0.044 |
| RCC01528 | *hypothetical protein* | Unknown Function | -1.67 | 0 |
| RCC02888 | *hypothetical protein* | Unknown Function | -1.67 | 0.02 |
| RCC01947 | *arginine translocator* | twin-arginine translocation pathway signal domain | -1.68 | 0.031 |
| RCC00534 | *hypothetical protein* | Unknown Function | -1.68 | 0.019 |
| RCC00855 | *sporulation domain-containing protein* | sporulation domain-containing protein | -1.7 | 0.011 |
| RCC02586 | *hypothetical protein* | Unknown Function | -1.7 | 0.015 |
| RCC02136 | *hypothetical protein* | Unknown Function | -1.7 | 0.035 |
| RCC02395 | *hypothetical protein* | Unknown Function | -1.71 | 0.013 |
| RCC00697 | *hypothetical protein* | Unknown Function | -1.72 | 0.001 |
| RCC00698 | *hypothetical protein* | Unknown Function | -1.72 | 0.008 |
| RCC02940 | *hypothetical protein* | Unknown Function | -1.72 | 0.042 |
| RCC00449 | *hypothetical protein* | Unknown Function | -1.73 | 0 |
| RCC02250 | *hypothetical protein* | Unknown Function | -1.75 | 0.012 |
| RCC01885 | *hypothetical protein* | Unknown Function | -1.76 | 0.039 |
| RCC01070 | *hypothetical protein* | Unknown Function | -1.76 | 0.036 |
| RCC02492 | *hypothetical protein* | Unknown Function | -1.77 | 0.036 |
| RCC00556 | *hypothetical protein* | Unknown Function | -1.78 | 0.007 |
| RCC00348 | *hypothetical protein* | Unknown Function | -1.81 | 0.022 |
| RCC02002 | *hypothetical protein* | Unknown Function | -1.81 | 0.014 |
| RCC01636 | *hypothetical protein* | Unknown Function | -1.83 | 0.007 |
| RCC00261 | *hypothetical protein* | Unknown Function | -1.84 | 0 |
| RCC00714 | *hypothetical protein* | Unknown Function | -1.84 | 0.008 |
| RCC00283 | *hypothetical protein* | Unknown Function | -1.89 | 0.012 |
| RCC00729 | *hypothetical protein* | Unknown Function | -1.91 | 0 |
| RCC01382 | *hypothetical protein* | Unknown Function | -1.91 | 0.01 |
| RCC00171 | *hypothetical protein* | Unknown Function | -1.93 | 0.006 |
| RCC00903 | *hypothetical protein* | Unknown Function | -1.99 | 0.009 |
| RCC02290 | *hypothetical protein* | Unknown Function | -2 | 0.012 |
| RCC01592 | *hypothetical protein* | Unknown Function | -2.02 | 0 |
| RCC02264 | *hypothetical protein* | Unknown Function | -2.05 | 0.003 |
| RCC01849 | *hypothetical protein* | Unknown Function | -2.08 | 0 |
| RCC01138 | *hypothetical protein* | Unknown Function | -2.1 | 0 |
| RCC01509 | *hypothetical protein* | Unknown Function | -2.11 | 0.006 |
| RCC01511 | *hypothetical protein* | Unknown Function | -2.74 | 0 |
| RCC02892 | *hypothetical protein* | Unknown Function | -2.86 | 0 |
| RCC02890 | *hypothetical protein* | Unknown Function | -3.01 | 0 |
| RCC00609 | *hypothetical protein* | Unknown Function | -3.1 | 0 |
| RCC00608 | *hypothetical protein* | Unknown Function | -4.09 | 0 |
|  |  |  |  |  |
| **COG T: Signal transduction mechanisms** | |  |  |  |
| RCC00584 | *anfA* | nitrogen fixation regulatory protein AnfA | 7.98 | 0 |
| RCC01495 | *fusA2* | translation elongation factor G | 3.46 | 0 |
| RCC01130 | *LuxR family* | LuxR family transcriptional regulator | 1.95 | 0 |
| RCC02197 | *AraC family* | two component AraC family transcriptional regulator | 1.89 | 0 |
| RCC00483 | *AsnC/Lrp family* | AsnC/Lrp family transcriptional regulator | 1.84 | 0 |
| RCC01415 | *serine/threonine-protein kinase* | serine/threonine protein kinase, bacterial | 1.72 | 0.001 |
| RCC03448 | *serine/threonine-protein phosphatase* | serine/threonine protein phosphatase 1 | 1.58 | 0 |
| RCC02431 | *dksA1* | DnaK suppressor protein | 1.56 | 0.02 |
| RCC03239 | *AsnC/Lrp family* | AsnC/Lrp family transcriptional regulator | 1.55 | 0.004 |
| RCC02790 | *CarD family* | CarD family transcriptional regulator | 1.51 | 0.003 |
| RCC02883 | *DeoR family* | K11531 lsr operon transcriptional repressor | 1.5 | 0.014 |
| RCC02928 | *XRE family* | XRE family transcriptional regulator | 1.5 | 0.007 |
| RCC00263 | *winged helix regulator* | winged helix family two component transcriptional regulator | 1.49 | 0.022 |
| RCC01566 | *LysR family* | LysR family transcriptional regulator | 1.49 | 0.043 |
| RCC01663 | *ctrA* | cell cycle transcriptional regulator CtrA | 1.42 | 0.019 |
| RCC02441 | *GntR family* | GntR family transcriptional regulator | 1.42 | 0.039 |
| RCC00873 | *XRE family* | XRE family transcriptional regulator | 1.38 | 0.031 |
| RCC00046 | *hvrA* | trans-acting regulatory protein HvrA | 1.36 | 0.031 |
| RCC00681 | *tspO* | signal transduction protein TspO | 1.3 | 0.044 |
| RCC01506 | *histidine kinase* | signal transduction histidine kinase | -1.27 | 0.044 |
| RCC00558 | *phosphodiesterase* | diguanylate cyclase/phosphodiesterase | -1.28 | 0.047 |
| RCC00763 | *hupT* | signal transduction histidine kinase HupT | -1.34 | 0.031 |
| RCC00346 | *phosphodiesterase* | diguanylate cyclase/phosphodiesterase | -1.37 | 0.05 |
| RCC02294 | *histidine kinase* | signal transduction histidine kinase | -1.4 | 0.042 |
| RCC01432 | *AraC family* | AraC family transcriptional regulator | -1.41 | 0.034 |
| RCC03068 | *cache sensor protein* | cache sensor protein | -1.46 | 0.01 |
| RCC00473 | *MerR family* | MerR family transcriptional regulator | -1.46 | 0.009 |
| RCC03005 | *phosphodiesterase* | diguanylate cyclase/phosphodiesterase | -1.47 | 0.018 |
| RCC02767 | *petR* | transcriptional regulator PetR | -1.5 | 0.01 |
| RCC02629 | *phosphodiesterase* | diguanylate cyclase/phosphodiesterase | -1.5 | 0.035 |
| RCC01902 | *TetR family* | TetR family transcriptional regulator | -1.51 | 0.042 |
| RCC02519 | *phosphodiesterase* | diguanylate cyclase/phosphodiesterase | -1.51 | 0.012 |
| RCC03301 | *phosphodiesterase* | diguanylate cyclase/phosphodiesterase | -1.52 | 0.002 |
| RCC00645 | *phosphodiesterase* | diguanylate cyclase/phosphodiesterase | -1.53 | 0.004 |
| RCC03324 | *rsbW* | anti-sigma regulatory factor | -1.53 | 0.035 |
| RCC00489 | *LysR family* | LysR family transcriptional regulator, regulator for m | -1.54 | 0.036 |
| RCC03025 | *dctS3* | C4-dicarboxylate transport sensor protein DctS | -1.55 | 0.05 |
| RCC02467 | *GntR family* | GntR family transcriptional regulator | -1.56 | 0.02 |
| RCC02374 | *LacI family* | LacI family transcriptional regulator, fructose operon | -1.56 | 0.001 |
| RCC01453 | *ArsR family* | ArsR family transcriptional regulator | -1.57 | 0.014 |
| RCC03322 | *GAF domain-containing protein* | GAF domain-containing protein | -1.59 | 0.019 |
| RCC00620 | *response regulator* | receiver modulated diguanylate cyclase/phosphodiesterase | -1.63 | 0.005 |
| RCC00902 | *XRE family* | XRE family transcriptional regulator | -1.64 | 0 |
| RCC02896 | *baeR* | transcriptional regulatory protein BaeR | -1.64 | 0.016 |
| RCC01431 | *AraC family* | AraC family transcriptional regulator | -1.64 | 0.018 |
| RCC02857 | *phosphodiesterase* | diguanylate cyclase/phosphodiesterase | -1.65 | 0.003 |
| RCC02249 | *AraC family* | AraC family transcriptional regulator | -1.69 | 0.007 |
| RCC00783 | *phosphodiesterase* | diguanylate cyclase/phosphodiesterase | -1.74 | 0 |
| RCC01619 | *Fis family* | Fis family transcriptional regulator | -1.74 | 0.011 |
| RCC01110 | *phosphodiesterase* | diguanylate cyclase/phosphodiesterase | -1.74 | 0.015 |
| RCC00440 | *zraR* | transcriptional regulatory protein ZraR | -1.75 | 0.003 |
| RCC01393 | *LuxR family* | LuxR family two component transcriptional regulator | -1.76 | 0.034 |
| RCC00621 | *histidine kinase* | signal transduction histidine kinase | -1.77 | 0.008 |
| RCC03026 | *dctR3* | C4-dicarboxylate transport transcriptional regulatory protein | -1.79 | 0.003 |
| RCC02546 | *winged helix regulator* | two-component system, OmpR famil | -1.8 | 0.001 |
| RCC01896 | *GntR family* | GntR family transcriptional regulator | -1.83 | 0.003 |
| RCC00537 | *response regulator* | receiver protein | -1.83 | 0.003 |
| RCC03147 | *MerR family* | MerR family transcriptional regulator | -1.9 | 0.001 |
| RCC03059 | *TetR family* | TetR family transcriptional regulator | -1.91 | 0 |
| RCC03298 | *LysR family* | LysR family transcriptional regulator | -2.01 | 0.001 |
| RCC01095 | *GntR family* | GntR family transcriptional regulator / MocR family am | -2.05 | 0.009 |
| RCC03064 | *HPP family/CBS domain-containing protein* | CBS domain-containing membrane protein | -2.76 | 0 |
|  |  |  |  |  |
|  |  |  |  |  |
| **COG U: Intracellular trafficking, secretion, and vesicular transport** | | |  |  |
| RCC01781 | *yajC* | preprotein translocase subunit YajC | 1.83 | 0 |
| RCC00029 | *secB* | protein-export chaperone SecB | 1.6 | 0.003 |
| RCC01458 | *tatA* | Sec-independent protein translocase TatA | 1.6 | 0.004 |
| RCC01940 | *hemolysin-type calcium-binding* | Predicted Function | 1.59 | 0.012 |
| RCC00285 | *secE* | preprotein translocase subunit SecE | 1.46 | 0 |
| RCC00322 | *secY* | preprotein translocase subunit SecY | 1.34 | 0.02 |
| RCC03312 | *lepB* | signal peptidase I | 1.33 | 0.028 |
| RCC00345 | *oxaA* | inner membrane protein OxaA | 1.32 | 0.033 |
| RCC00220 | *secA* | preprotein translocase subunit SecA | 1.28 | 0.022 |
| RCC03159 | *hemolysin-type calcium-binding* | Predicted Function | -1.44 | 0.004 |
| RCC02066 | *hemolysin D* | HlyD family secretion protein | -1.63 | 0.011 |
| RCC01603 | *hemolysin-III family protein* | K11068 hemolysin III | -1.69 | 0.001 |
| RCC02520 | *hemolysin-type calcium-binding* | Predicted Function | -1.75 | 0.004 |
| RCC03392 | *hemolysin D* | HlyD family secretion protein | -1.88 | 0 |
| RCC01456 | *MarC family membrane protein* | multiple antibiotic resistance protein | -1.98 | 0.004 |
|  |  |  |  |  |
| **COG V: Defense mechanisms** | |  |  |  |
| RCC00103 | *hemolysin-type calcium-binding* | peptide/nickel transport system substr | 1.88 | 0 |
| RCC01926 | *rnhA1* | ribonuclease H | 1.76 | 0.002 |
| RCC01930 | *hsdM3* | type I restriction-modification system RcaSBIV subunit M | 1.4 | 0.009 |
| RCC00183 | *ABC transporter ATP-binding* | K12541 ATP-binding cassette, subfamily C, bacteri | -1.26 | 0.04 |
| RCC02898 | *ABC transporter ATP-binding* | ATP-binding cassette, subfamily B, bacteri | -1.51 | 0.01 |
| RCC00615 | *acrA* | acriflavine resistance protein A | -1.51 | 0.004 |
| RCC02065 | *HlyB family* | secretion ATP-binding protein, HlyB family | -1.52 | 0.017 |
| RCC02457 | *lolD2* | lipoprotein-releasing system ATP-binding protein | -1.73 | 0.003 |
|  |  |  |  |  |
| **COG X: Photosynthesis** | |  |  |  |
| RCC02533 | *pucDE* | light-harvesting protein B-800/850 subunit gamma | 1.55 | 0.002 |
| RCC00691 | *pufB* | light-harvesting protein B-870 subunit beta | 1.53 | 0.003 |
| RCC00692 | *pufA* | light-harvesting protein B-870 subunit alpha | 1.5 | 0.004 |
| RCC00686 | *bchC* | bacteriochlorophyllide A dehydrogenase | 1.39 | 0.03 |
| RCC00669 | *bchE* | magnesium-protoporphyrin IX monomethyl estercyclase | 1.35 | 0.019 |
| RCC00659 | *puhA* | photosynthetic reaction center subunit H | 1.33 | 0.03 |
| RCC00661 | *bchM* | magnesium-protoporphyrin O-methyltransferase | -1.25 | 0.034 |
| RCC00670 | *bchJ* | bacteriochlorophyll 4-vinyl reductase | -1.39 | 0.001 |
| RCC00675 | *bchO* | magnesium-chelatase BchO | -1.52 | 0.01 |
| RCC00676 | *bchD* | magnesium chelatase ATPase subunit D | -1.61 | 0.007 |
| Adjusted p value cutoff was set to 0.05. | |  |  |  |
