# Supplementary material for: Analysis of the FnrL regulon in Rhodobacter capsulatus reveals limited regulon overlap with orthologues from Rhodobacter sphaeroides and Escherichia coli
Source: BMC Genomics. 2015 Nov 4;16:895. doi: 10.1186/s12864-015-2162-4 (PMC4634722; doi:10.1186/s12864-015-2162-4)
Supplement: Additional file 3: Table S3. — FnrL ChIP-seq signal with corresponding p-value > 0.05 based on RNA-seq expression change. (DOCX 66 kb) [file 12864_2015_2162_MOESM3_ESM.docx]

| **Table S3:** FnrL ChIP-seq signal with corresponding p-value > 0.05 based on RNA-seq expression change | | | | |  |
| --- | --- | --- | --- | --- | --- |
| **Locus ID** | **Gene Name** | **Description** | **Recognition Sequence** | **P-value** | **Fold Change** |
| RCAP_rcc00022 | *Oxidoreductase* | oxidoreductase | ATGATTTACCGCAA | 0.08 | 1.38 |
| RCAP_rcc01724 | *speB1* | agmatinase | TTGATCTGCGTCAA | 0.08 | 1.33 |
| RCAP_rcc03523 | *flbT* | flagellin synthesis repressor protein FlbT | CTGATCGACATCAA | 0.07 | -2.14 |
| RCAP_rcc01027 | *hypothetical protein* | hypothetical protein | TTGACCAAGGTCAA | 0.08 | -1.64 |
| RCAP_rcc02988 | *hypothetical protein* | hypothetical protein | TTGACCCAGATCAA | 0.051 | -1.41 |
