# Supplementary material for: Analysis of the FnrL regulon in Rhodobacter capsulatus reveals limited regulon overlap with orthologues from Rhodobacter sphaeroides and Escherichia coli
Source: BMC Genomics. 2015 Nov 4;16:895. doi: 10.1186/s12864-015-2162-4 (PMC4634722; doi:10.1186/s12864-015-2162-4)
Supplement: Additional file 4: Table S4. — FnrL predicted binding site based on PWM with corresponding RNA-seq expression change. (DOCX 165 kb) [file 12864_2015_2162_MOESM4_ESM.docx]

| **Table S4:** FnrL predicted* binding site based on PWM with corresponding RNA-seq expression change | | | | | |
| --- | --- | --- | --- | --- | --- |
| **Locus ID** | **Gene Name** | **Description** | **Recognition Sequence** | **Regulation** | **Fold Change** |
| **COG C: Energy production and conversion** | | |  |  |  |
| *RCC02845 | *torA* | trimethylamine-N-oxide reductase | CTGACCGGGGTCAA | + | 2.99 |
| *RCC01729 | *oxidoreductase* | oxidoreductase | ATGATCCAAGTCAT | + | 2.69 |
| *RCC02791 | *fdxA* | ferredoxin II | GTGATCACGATCAA | + | 1.89 |
| *RCC00588 | *anfK* | nitrogenase iron-iron protein subunit beta | GTGATGGAGGTCAA | + | 1.54 |
| *RCC00720 | *sucC* | succinyl-CoA synthetase (ADP-forming) subunit beta | ATGCTGGAGATCAA | + | 1.48 |
| *RCC00744 | *atpF* | ATP synthase F0 subunit B | CTGATCAAGTTCAA | + | 1.23 |
| *RCC00544 | *aldehyde dehydrogenase* | aldehyde dehydrogenase (NAD+) | GTGATCCAGACCAA | - | -1.28 |
| *RCC03507 | *gpsA* | glycerol-3-phosphate dehydrogenase | ATGACGTTTTTCAA | - | -1.34 |
| *RCC00831 | *dld* | D-lactate dehydrogenase | TTGAACATCCTCAA | - | -1.38 |
| **COG D: Cell cycle control, cell division, chromosome partitioning** | | |  |  |  |
| *RCC03167 | *hypothetical protein* | cell division transport system permease protein | GTGATGCGGTTCAA | - | -1.5 |
| **COG E: Amino acid transport and metabolism** | | |  |  |  |
| *RCC01214 | *ilvC* | ketol-acid reductoisomerase | GTGAACCTGATCAA | + | 1.67 |
| *RCC03387 | *glnB2* | nitrogen regulatory protein P-II | GTGACGGAAATCAA | + | 1.65 |
| *RCC02357 | *trpG* | anthranilate synthase component II | ATGAGCGAACTCAA | + | 1.56 |
| *RCC01674 | *glnA3* | glutamine synthetase | ATGAGCGCAGTCAA | + | 1.52 |
| *RCC00341 | *argB* | acetylglutamate kinase | CTGAAGGCGGTCAA | + | 1.48 |
| *RCC00553 | *argC* | N-acetyl-gamma-glutamyl-phosphate reductase | CTGATGCCCTTCAA | + | 1.44 |
| *RCC00082 | *leuD* | 3-isopropylmalate dehydratase small subunit | CTGAAAACGATCAA | + | 1.42 |
| *RCC02452 | *potD4* | polyamine ABC transporter | ATGATGCTGGTCAA | + | 1.4 |
| *RCC00484 | *ald* | alanine dehydrogenase | GTGAAAGAGATCAA | + | 1.37 |
| *RCC02787 | *metB* | O-acetylhomoserine aminocarboxypropyltransferase | GTGAAATTGGTCAA | + | 1.35 |
| *RCC03001 | *proA* | glutamate-5-semialdehyde dehydrogenase | CTGACCTCGTTCAA | + | 1.33 |
| *RCC01363 | *leuA* | 2-isopropylmalate synthase | GTGATGGCGATCAA | + | 1.21 |
| *RCC01021 | *ugpC* | sn-glycerol-3-phosphate transport ATP-binding protein | GTGATAAACCTCAA | - | -1.55 |
| *RCC00797 | *hisG* | ATP phosphoribosyltransferase catalytic subunit | ATGAGCGCGATCAA | - | -1.67 |
| **COG F: Nucleotide transport and metabolism** | | |  |  |  |
| *RCC01462 | *nrdJ2* | ribonucleoside-diphosphate reductase NrdJ | CTGAAACGCGTCAA | + | 1.41 |
| **COG G: Carbohydrate transport and metabolism** | | |  |  |  |
| *RCC02656 | *hypothetical protein* | hypothetical protein | CTGACCAAGCTCAA | + | 1.58 |
| *RCC01715 | *eno* | phosphopyruvate hydratase | ATGAACATCATCAA | + | 1.42 |
| *RCC02373 | *monosacharide ABC transporter* | D-xylose transport s | GTGACCTTCCTCAA | + | 1.41 |
| *RCC01774 | *aglA* | alpha-glucosidase | CTGATGTGCCTCAA | + | 1.32 |
| *RCC00014 | *xylA* | xylose isomerase | ATGACCAAGCTCAA | + | 1.28 |
| *RCC00077 | *gpmI* | 2,3-bisphosphoglycerate-independent phosphoglycerate | CTGATCATCGTCAA | + | 1.27 |
| *RCC00577 | *cbbQ* | Putative RubisCO regulator CbbQ protein | ATGAAGGATCTCAA | - | -1.31 |
| *RCC00054 | *inositol monophosphatase* | myo-inositol-1(or 4)-monophosphatase | ATGACTTAAATCAA | - | -1.34 |
| **COG H: Coenzyme transport and metabolism** | | |  |  |  |
| *RCC00195 | *hemH* | ferrochelatase | GTGATCGTCATCAA | + | 1.34 |
| *RCC02038 | *cobF* | precorrin-6A synthase | CTGATCCCGCTCAA | - | -1.39 |
| *RCC00712 | *moaA1* | molybdenum cofactor biosynthesis protein A | GTGACCTGAATCAA | - | -1.42 |
| *RCC02430 | *nahG* | salicylate hydroxylase | CTGAAGATGCTCAA | - | -1.73 |
| **COG I: Lipid transport and metabolism** | | |  |  |  |
| *RCC01634 | *fabZ* | (3R)-hydroxymyristoyl-ACP dehydratase | CTGAGCCTGATCAA | + | 1.71 |
| *RCC00746 | *phbC* | poly(3-hydroxyalkanoate) polymerase | ATGACGCAGATCAA | + | 1.56 |
| *RCC01791 | *cyclase* | cyclase/dehydrase | GTGATCTCGTTCAA | + | 1.53 |
| *RCC02669 | *fabA* | 3-hydroxydecanoyl-ACP dehydratase | CTGACCGGCTTCAA | + | 1.31 |
| *RCC03449 | *atoB3* | acetyl-CoA acetyltransferase | GTGATCGAAAGCAA | - | -1.45 |
| *RCC01512 | *mccB* | methylcrotonoyl-CoA carboxylase subunit beta | GTGAGCCACCTCAA | - | -2.3 |
| **COG J: Translation, ribisomal tructure and biogenesis** | | |  |  |  |
| *RCC00309 | *rpsQ* | 30S ribosomal protein S17 | CTGAACCAGTTCAA | + | 1.48 |
| *RCC03416 | *endoribonuclease L-PSP family protein* | endoribonuclease L-PSP family protein | CTGAGCGCGCTCAA | + | 1.42 |
| *RCC03490 | *endoribonuclease, L-PSP family* | endoribonuclease, L-PSP family | ATGACTCGTGTCAA | + | 1.4 |
| *RCC00301 | *rplW* | 50S ribosomal protein L23 | GTGAAGGCGGTCAA | + | 1.35 |
| *RCC00312 | *rplE* | 50S ribosomal protein L5 | TTGAAGGCTTTCAA | + | 1.34 |
| *RCC00854 | *argS* | arginyl-tRNA synthetase | GTGAAGCTCTTCAA | + | 1.24 |
| *RCC00041 | *hypothetical protein* | tRNA threonylcarbamoyladenosine biosynthesis protein | GTGATGACGCTCAA | - | -1.28 |
| **COG K: Transcription** | |  |  |  |  |
| *RCC00326 | *rpoA* | DNA-directed RNA polymerase subunit alpha | CTGAACGAGATCAA | + | 1.31 |
| *RCC03147 | *MerR family transcriptional regulator* | MerR family transcriptional regulator | GTGATCTGGGTCAA | - | -1.91 |
| *RCC03059 | *TetR family transcriptional regulator* | TetR family transcriptional regulator | CTGACCGGGGTCAA | - | -1.92 |
| **COG L: Replication, recombination and repair** | | |  |  |  |
| *RCC00996 | *hup2* | DNA-binding protein HU | CTGACGCTGTTCAA | + | 1.43 |
| *RCC01751 | *recA* | RecA protein | GTGATCTTCATCAA | + | 1.21 |
| *RCC00038 | *addB* | double-strand break repair protein AddB | CTGATCGCCTTCAA | - | -1.23 |
| *RCC01276 | *Cas1 family CRISPR-associated protein* | CRISP-associated protein Cas1 | GTGAGCCTCTTCAA | - | -1.39 |
| *RCC02007 | *phage integrase* | phage integrase | CTGACGGCGGTCAA | - | -1.47 |
| **COG M: Cell wall/membrane/envelope biogenesis** | | |  |  |  |
| *RCC01873 | *peptidoglycan-binding domain 1* | peptidoglycan-binding domain 1 protein | ATGATGGTGATCAA | + | 1.37 |
| *RCC01550 | *M23 family peptidase* | M23 family peptidase | ATGACCTTCATCAA | + | 1.28 |
| *RCC00269 | *lipoprotein* | lipoprotein | CTGTTCAACATCAA | - | -1.44 |
| *RCC02627 | *dacB* | penicillin-binding protein 4 | ATGAAGCCGTTCAA | - | -1.49 |
| *RCC01637 | *lpxB* | lipid-A-disaccharide synthase | GTGACGCTGGTCAA | - | -1.53 |
| *RCC01115 | *lipoprotein* | lipoprotein | CTGAGCACGCTCAA | - | -1.54 |
| **COG N: Cell motility** | |  |  |  |  |
| *RCC02591 | *surface presentation of antigens* | flagellar motor switch protein FliM | TTGATTCAGGTCAA | - | -1.27 |
| *RCC00010 | *flgI* | flagellar P-ring protein FlgI | CTGAAAAGCATCAA | - | -1.51 |
| *RCC02611 | *mcpA3* | methyl-accepting chemotaxis protein McpA | CTGAGCGAGGTCAA | - | -1.64 |
| **COG O: Post-translational modification, protein turnover, and chaperones** | | |  |  |  |
| *RCC02478 | *groL* | chaperonin GroL | CTGAAAGGCGTCAA | + | 1.56 |
| *RCC03257 | *msrA1* | peptide-methionine-(S)-S-oxide reductase | GTGATGATCGTCAA | + | 1.55 |
| *RCC02460 | *cspA2* | cold shock protein CspA | GTGAAATGGTTCAA | + | 1.45 |
| *RCC00036 | *trxA1* | thioredoxin | GTGAAGATCGTCAA | + | 1.36 |
| *RCC03141 | *cspD* | cold shock-like protein CspD | GTGAAATGGTTCAA | + | 1.27 |
| *RCC01786 | *ccmB* | heme exporter protein B | TTGATGCGAGTCAA | - | -1.36 |
| *RCC02865 | *pflA2* | [pyruvate formate-lyase]-activating enzyme | ATGCTCGACATCAA | - | -1.38 |
| *RCC02893 | *cspA3* | cold shock protein CspA | GTGAAATGGTTCAA | - | -1.66 |
| *RCC00607 | *cspA1* | cold shock protein CspA | GTGAAGTTTTTCAA | - | -1.77 |
| *RCC00806 | *heat shock protein* | DnaJ like chaperone protein | GTGATCGCGCTCAA | - | -1.87 |
| **COG P: Inorganic ion transport** | |  |  |  |  |
| *RCC02843 | *moaA2* | molybdenum cofactor biosynthesis protein A | GTGAAGCGGGTCAA | + | 1.6 |
| *RCC02521 | *pyrimidine ABC transporter* | NitT/TauT family transport | CTGAAGCTTGTCAA | + | 1.36 |
| *RCC03015 | *kefC2* | glutathione-regulated potassium-efflux system | ATGACCGGGCTCAA | - | -1.65 |
| **COG R: General function prediction only** | | |  |  |  |
| *RCC00938 | *phage terminase large subunit* | phage terminase large subunit | GTGATCGCCATCAA | + | 2.73 |
| *RCC02612 | *transthyretin family protein* | 5-hydroxyisourate hydrolase | CTGATCCGGCTCAA | + | 1.51 |
| *RCC02463 | *hemolysin-type protein* | hemolysin-type calcium-binding repeat family protein | GTGAACTACATCAA | + | 1.39 |
| *RCC02119 | *type 12 family methyltransferase* | type 12 family methyltransferase | ATGAAGCCTATCAA | + | 1.38 |
| *RCC02072 | *nonfunctional major facilitator protein* | nonfunctional major facilitator superfamily protein | GTGATGGCGGTCAA | - | -1.37 |
| *RCC03159 | *hemolysin-type protein* | hemolysin-type calcium-binding repeat family protein | ATGATCACCGTCAA | - | -1.44 |
| *RCC00178 | *hemolysin-type protein* | hemolysin-type calcium-binding repeat family protein | CTGATCGGCAGCAA | - | -1.47 |
| *RCC01687 | *protein* | HK97 family phage major capsid protein | CTGAAGGAAGTCAA | - | -1.62 |
| **COG S: Function unknown** | |  |  |  |  |
| *RCC03304 | *hypothetical protein* | hypothetical protein | TTGAACAAAATCAA | + | 2.39 |
| *RCC01423 | *hypothetical protein* | hypothetical protein | ATGAAAACGATCAA | + | 1.61 |
| *RCC01038 | *hypothetical protein* | hypothetical protein | CTGATCGCCTTCAA | + | 1.58 |
| *RCC02939 | *hypothetical protein* | hypothetical protein | TTGACGATTCTCAA | + | 1.56 |
| *RCC00935 | *hypothetical protein* | hypothetical protein | TTGATCGAGACCAA | + | 1.55 |
| *RCC03379 | *hypothetical protein* | hypothetical protein | CTGAGGGTCATCAA | + | 1.4 |
| *RCC02750 | *hypothetical protein* | hypothetical protein | ATGAGTTTCGTCAA | + | 1.37 |
| *RCC02343 | *hypothetical protein* | hypothetical protein | CTGAGCGGCATCAA | + | 1.27 |
| *RCC02433 | *hypothetical protein* | hypothetical protein | CTGATCTGTCTCAA | - | -1.3 |
| *RCC01064 | *hypothetical protein* | hypothetical protein | GTGACGGCGATCAA | - | -1.47 |
| *RCC00499 | *hypothetical protein* | hypothetical protein | ATGAAGTTCTTCAA | - | -1.62 |
| *RCC01885 | *hypothetical protein* | hypothetical protein | CTGAAGGCGCTCAA | - | -1.77 |
| *RCC02892 | *hypothetical protein* | hypothetical protein | TTGACGGCCCTCAA | - | -2.87 |
| **COG T: Signal transduction mechanisms** | | |  |  |  |
| *RCC01415 | *serine/threonine-protein kinase* | serine/threonine protein kinase, bacterial | ATGAAAGTGATCAA | + | 1.72 |
| *RCC03448 | *serine/threonine-protein phosphatase* | serine/threonine protein phosphatase 1 | CTGATCGTGCTCAA | + | 1.58 |
| *RCC00046 | *hvrA* | trans-acting regulatory protein HvrA | CTGAACGAACTCAA | + | 1.37 |
| *RCC02540 | *diguanylate cyclase/phosphodiesterase* | diguanylate cyclase/phosphodiesterase | GTGATCTCGGTCAA | - | -1.32 |
| *RCC02896 | *baeR* | response regulatory protein BaeR | GTGCTCGACATCAA | - | -1.65 |
| *RCC01110 | *diguanylate cyclase* | diguanylate cyclase/phosphodiesterase | CTGAGCGAGATCAA | - | -1.74 |
| *RCC00783 | *diguanylate cyclase/phosphodiesterase* | diguanylate cyclase/phosphodiesterase | CTGATGTCGATCAA | - | -1.74 |
| **COG U: Intracellular trafficking, secretion, and vesicular transport** | | |  |  |  |
| *RCC01458 | *tatA* | Sec-independent protein translocase TatA | CTGACAAGGTTCAA | + | 1.6 |
| *RCC00285 | *secE* | preprotein translocase subunit SecE | ATGACGGTGGTCAA | + | 1.46 |
| *RCC03312 | *lepB* | signal peptidase I | GTGCTGCAGATCAA | + | 1.34 |
| **COG V: Defense Mechanism** | |  |  |  |  |
| *RCC01286 | *mcrB* | McrBC restriction endonuclease system subunit McrB | GTGATGCGCTTCAA | + | 1.28 |
