# Supplementary material for: Analysis of the FnrL regulon in Rhodobacter capsulatus reveals limited regulon overlap with orthologues from Rhodobacter sphaeroides and Escherichia coli
Source: BMC Genomics. 2015 Nov 4;16:895. doi: 10.1186/s12864-015-2162-4 (PMC4634722; doi:10.1186/s12864-015-2162-4)
Supplement: Additional file 5: Table S5. — Clusters of orthologous groups definitions. (DOCX 66 kb) [file 12864_2015_2162_MOESM5_ESM.docx]

| **Table S5:** Clusters of orthologous groups definitions (COGs) | |
| --- | --- |
| **Cellular Processing and Signaling** | |
| D | Cell cycle control, cell division, chromosome partitioning |
| M | Cell wall/membrane/envelope biogenesis |
| N | Cell motility |
| O | Post-translational modification, protein turnover, and chaperones |
| T | Signal transduction mechanisms |
| U | Intracellular trafficking, secretion, and vesicular transport |
| V | Defense mechanisms |
| W | Extracellular structures |
| Y | Nuclear structure |
| Z | Cytoskeleton |
| **Information Storage and Processing** | |
| A | RNA processing and modification |
| B | Chromatin structure and dynamics |
| J | Translation, ribosomal structure and biogenesis |
| K | Transcription |
| L | Replication, recombination and repair |
| **Metabolism** | |
| X | Photosynthesis |
| C | Energy production and conversion |
| E | Amino acid transport and metabolism |
| F | Nucleotide transport and metabolism |
| G | Carbohydrate transport and metabolism |
| H | Coenzyme transport and metabolism |
| I | Lipid transport and metabolism |
| P | Inorganic ion transport and metabolism |
| Q | Secondary metabolites biosynthesis, transport, and catabolism |
| **Poorly Characterized** | |
| R | General function prediction only |
| S | Function unknown |
